# On the Efficiency of All-Pay Mechanisms

**DOI:** 10.1007/s00453-017-0296-2

**Published:** 2017-03-17

**Authors:** George Christodoulou, Alkmini Sgouritsa, Bo Tang

**Affiliations:** 1 Department of Computer Science, University of Liverpool, Liverpool, UK; 2 Department of Computer Science, Oxford University, Oxford, UK

**Keywords:** Nash equilibrium, Price of anarchy, All-pay auction

## Abstract

We study the inefficiency of mixed Nash equilibria, expressed as the price of anarchy, of all-pay auctions in three different environments: combinatorial, multi-unit and single-item auctions. First, we consider item-bidding combinatorial auctions where *m* all-pay auctions run in parallel, one for each good. For fractionally subadditive valuations, we strengthen the upper bound from 2 (Syrgkanis and Tardos in Proceedings of the 45th symposium on theory of computing (STOC ’13), 2013) to 1.82 by proving some structural properties that characterize the mixed Nash equilibria of the game. Next, we design an all-pay mechanism with a randomized allocation rule for the multi-unit auction. We show that, for bidders with submodular valuations, the mechanism admits a unique, $75\%$ efficient, pure Nash equilibrium. The efficiency of this mechanism outperforms all the known bounds on the price of anarchy of mixed Nash equilibria in mechanisms used for multi-unit auctions. Finally, we analyze single-item all-pay auctions motivated by their connection to contests and show tight bounds on the price of anarchy with respect to social welfare, revenue and maximum bid.

## Introduction

It is a common economic phenomenon in competitions that agents make irreversible investments without knowing the outcome. *All-pay* auctions are widely used in economics to capture such situations, where all players, even the losers, pay their bids. For example, a lobbyist can make a monetary contribution in order to influence decisions made by the government. Usually the group invested the most increases their winning chances, but all groups have to pay regardless of the outcome. In addition, all-pay auctions have been shown useful to model rent seeking, political campaigns and R&D races. There is a well-known connection between all-pay auctions and *contests* [21]. In particular, the all-pay auction can be viewed as a single-prize contest, where the payments correspond to the effort that players make in order to win the competition.

In this paper, we study the efficiency of mixed Nash equilibria in all-pay auctions with complete information, from a worst-case analysis perspective, using the *price of anarchy* [16] as a measure. As social objective, we consider the *social welfare*, i.e. the sum of the bidders’ valuations. We study the equilibria induced from all-pay mechanisms in three fundamental resource allocation scenarios; combinatorial auctions, multi-unit auctions and single-item auctions.

In a combinatorial auction, a set of items are allocated to a group of selfish individuals. Each player has different preferences for different subsets of the items and this is expressed via a *valuation set* function. A multi-unit auction can be considered as an important special case, where there are multiple copies of a single good. Hence the valuations of the players are not set functions, but depend only on the number of copies received. Multi-unit auctions have been extensively studied since the seminal work by Vickrey [24]. As already mentioned, all-pay auctions have received a lot of attention for the case of a single item, as they model all-pay contests and procurements via contests.

### Contribution

*Combinatorial Auctions* Our first result is on the price of anarchy of simultaneous all-pay auctions with item-bidding that was previously studied by Syrgkanis and Tardos [23]. For fractionally subadditive valuations, it was previously shown that the price of anarchy was at most 2 [23] and at least $e/(e-1)\approx 1.58$ [8]. We narrow further this gap, by improving the upper bound to 1.82. In order to obtain the bound, we come up with several structural theorems that characterize mixed Nash equilibria in simultaneous all-pay auctions.

*Multi-Unit Auctions* Our next result shows a novel use of all-pay mechanisms to the multi-unit setting. We propose an all-pay mechanism with a randomized allocation rule inspired by Kelly’s seminal proportional-share allocation mechanism [15]. We show that this mechanism admits a *unique*, $75\%$ efficient *pure* Nash equilibrium and no other mixed Nash equilibria exist, when bidders’ valuations are submodular. As a consequence, the price of anarchy of our mechanism outperforms all current price of anarchy bounds of mixed Nash equilibria in prevalent multi-unit auctions including uniform price auction [18] and discriminatory auction [14], where the bound is $e/(e-1)\approx 1.58$.

*Single-Item Auctions* Finally, we study the efficiency of a single-prize contest that can be modeled as a single-item all-pay auction. We show a tight bound on the price of anarchy for mixed Nash equilibria which is approximately 1.185. By following previous study on the procurement via contest, we further study two other standard objectives, *revenue* and *maximum bid*. We evaluate the performance of all-pay auctions in the prior-free setting, i.e. no distribution over bidders’ valuation is assumed. We show that both the revenue and the maximum bid of any mixed Nash equilibrium are at least as high as $v_2/2$, where $v_2$ is the second highest valuation. In contrast, the revenue and the maximum bid in some mixed Nash equilibrium may be less than $v_2/2$ when using reward structure other than allocating the entire reward to the highest bidder. This result coincides with the optimal crowdsourcing contest developed in [6] for the setting with prior distributions. We also show that in conventional procurements (modeled by first-price auctions), $v_2$ is exactly the revenue and maximum bid in the worst equilibrium. So procurement via all-pay contests is a 2-approximation to the conventional procurement in the context of worst-case equilibria.

### Related Work

The inefficiency of Nash equilibria in auctions has been a well-known fact (see e.g. [17]). Existence of efficient equilibria of simultaneous sealed bid auctions in full information settings was first studied by Bikhchandani [3]. Christodoulou et al.  [7] initiated the study of the (Bayesian) price of anarchy of simultaneous auctions with item-bidding. Several variants have been studied since then [2, 10–12], as well as multi-unit auctions [14, 18]. Recently, Feldman, Lucier and Nisan showed that, in first-price single-item auctions, correlated equilibria are always efficient and the price of anarchy of coarse equilibria is exactly $e/(e-1)$.

Syrgkanis and Tardos [23] proposed a general smoothness framework for several types of mechanisms and applied it to settings with fractionally subadditive bidders obtaining several upper bounds (e.g., first price auction, all-pay auction, and multi-unit auction). Christodoulou et al. [8] constructed tight lower bounds for first-price auctions and showed a tight price of anarchy bound of 2 for all-pay auctions with subadditive valuations. Roughgarden [20] presented an elegant methodology to provide price of anarchy lower bounds via a reduction from the hardness of the underlying optimization problems.

All-pay auctions and contests have been studied extensively in economic theory. Baye et al. [1], fully characterized the Nash equilibria in single-item all-pay auction with complete information. The connection between all-pay auctions and crowdsourcing contests was proposed in [9]. Chawla et al. [6] studied the design of optimal crowdsourcing contest to optimize the maximum bid in all-pay auctions when agents’ value are drawn from a specific distribution independently.

## Preliminaries

In a *combinatorial auction*, *n* *players* compete on *m* *items*. Every player (or *bidder*) $i\in [n]$ has a valuation function $v_i: \{0,1\}^m \rightarrow {\mathbb {R}}^+$ which is monotone and normalized, that is, $\forall S \subseteq T \subseteq [m]$, $v_i(S)\le v_i(T),$ and $v_i(\emptyset )=0.$ The outcome of the auction is represented by a tuple of $(\mathbf {X},\mathbf {p})$ where $\mathbf {X}=(X_1,\ldots , X_n)$ specifies the allocation of items ($X_i$ is the set of items allocated to player *i*) and $\mathbf {p}=(p_1,\ldots ,p_n)$ specifies the buyers’ payments ($p_i$ is the payment of player *i* for the allocation $\mathbf {X}$). In the *simultaneous item-bidding* auction, every player $i\in [n]$ submits a non-negative bid $b_{ij}$ for each item $j\in [m].$ The items are then allocated by independent auctions, i.e. the allocation and payment rule for item *j* only depend on the players’ bids on item *j*. In a simultaneous *all-pay* auction the allocation and payment for each player is determined as follows: each item $j \in [m]$ is allocated to the bidder $i^*$ with the highest bid for that item, i.e. $i^* = \arg \max _{i}b_{ij}$, and each bidder *i* is charged an amount equal to $p_{i}=\sum _{j\in [m]}b_{ij}$. It is worth mentioning that, for any bidder profile, there always exists a tie-breaking rule such that mixed Nash equilibria exist [22].

### Definition 1

*(Valuations)* Let $v: 2^{[m]}\rightarrow {\mathbb {R}}$ be a valuation function. Then *v* is called *(a) additive*, if $v(S)=\sum _{j\in S} v(\{j\});$ *(b) submodular*, if $v(S\cup T)+v(S\cap T)\le v(S)+v(T);$ *(c) fractionally subadditive* or *XOS*, if *v* is determined by a finite set of additive valuations $\xi _k$ such that $v(S)= \max _{k} \xi _k(S)$.

The classes of the above valuations are in increasing order of inclusion.

*Multi-Unit Auction* In a multi-unit auction, *m* copies of an item are sold to *n* bidders. Here, bidder *i* ’s valuation is a function that depends on the number of copies he gets. That is $v_i: \{0, 1,\dots , m\} \rightarrow {\mathbb {R}}^+$ and it is non-decreasing and normalized, with $v_i(0) = 0$. We say a valuation $v_i$ is *submodular*, if it has non-increasing marginal values, i.e. $v_i(s+1)-v_i(s)\ge v_i(t+1)-v_i(t)$ for all $s\le t$.

*Nash Equilibrium and Price of Anarchy* We use $b_i$ to denote a pure strategy of player *i* which might be a single value or a vector, depending on the auction. So, for the case of *m* simultaneous auctions, $b_i=(b_{i1},\ldots ,b_{im})$. We denote by $\mathbf {b}_{-i}=(b_1,\ldots ,b_{i-1},b_{i+1},\ldots ,b_n)$ the strategies of all players except for *i*. Any *mixed strategy* $B_i$ of player *i* is a probability distribution over pure strategies.

For any profile of strategies, $\mathbf {b}=(b_1,\ldots ,b_n)$, $\mathbf {X}(\mathbf {b})$ denotes the allocation under the strategy profile $\mathbf {b}$. The valuation of player *i* for the allocation $\mathbf {X}(\mathbf {b})$ is denoted by $v_i(\mathbf {X}(\mathbf {b}))=v_i(\mathbf {b})$. The *utility* $u_i$ of player *i* is defined as the difference between her valuation and payment: $u_i(\mathbf {X}(\mathbf {b}))=u_i(\mathbf {b})=v_i(\mathbf {b})-p_i(\mathbf {b})$.

### Definition 2

*(Nash equilibrium)* A bidding profile $\mathbf {b}=(b_1,\ldots ,b_n)$ forms a pure Nash equilibrium if for every player *i* and all bids $b'_i$, $u_i(\mathbf {b})\ge u_i(b'_i, \mathbf {b}_{-i})$. Similarly, a mixed bidding profile $\mathbf {B}=\times _i B_i$ is a mixed Nash equilibrium if for all bids $b'_i$ and every player *i*, $\mathbb {E}_{\mathbf {b}\sim \mathbf {B}}[u_i(\mathbf {b})]\ge \mathbb {E}_{\mathbf {b}_{-i}\sim \mathbf {B}_{-i}}[u_i(b'_i,\mathbf {b}_{-i})]$. Clearly, any pure Nash equilibrium is also a mixed Nash equilibrium.

Our global objective is to maximize the sum of the valuations of the players for their received allocations, i.e., to maximize the *social welfare* $SW(\mathbf {X})=\sum _{i\in [n]} v_i(X_i).$ So $\mathbf {O}(\mathbf {v})=\mathbf {O}=(O_1,\ldots ,O_n)$ is an *optimal allocation* if $SW(\mathbf {O})=\max _{\mathbf {X}} SW(\mathbf {X})$. In Sect. 5, we also study two other objectives: the *revenue*, which equals the sum of the payments, $\sum _i p_i$, and the *maximum payment*, $\max _i b_i$. We also refer to the maximum payment as the *maximum bid*.

### Definition 3

*(Price of anarchy)* Let ${{\mathcal {I}}}$ be the set of all instances, i.e. ${\mathcal {I}}$ includes the instances for every set of bidders and items and any possible valuation functions. The mixed price of anarchy, PoA, of a mechanism is defined as

$$\begin{aligned} \hbox {PoA} = \max _{I \in {\mathcal {I}}} \max _{\mathbf {B}\in {\mathcal {E}}(I)} \frac{SW(\mathbf{O})}{\mathbb {E}_{\begin{array}{c} \mathbf {b}\sim \mathbf {B} \end{array}}[SW(\mathbf {X}(\mathbf {b}))]}, \end{aligned}$$

where ${\mathcal {E}}(I)$ is the class of mixed Nash equilibria for the instance $I \in {\mathcal {I}}$. The pure PoA is defined as above but restricted in the class of pure Nash equilibria.

Let $\mathbf {B}=(B_1,\ldots ,B_n)$ be a profile of mixed strategies. Given the rofile $\mathbf {B}$, we fix the notation for the following *cumulative distribution functions (CDF):* $G_{ij}$ is the CDF of the bid of layer *i* for item *j*;  $F_{j}$ is the CDF of the highest bid for tem *j* and $F_{ij}$ is the CDF of the highest bid for item *j* if we exclude the bid of player *i*. Observe that $F_j=\prod _k G_{kj}$ and $F_{ij}=\prod _{k\ne i} G_{kj}.$ We also use $\varphi _{ij}(x)$ to denote the probability that player *i* gets item *j* by bidding *x*. Then, $\varphi _{ij}(x)\le F_{ij}(x).$ When we refer to a single item, we may drop the index *j*. Whenever it is clear from the context, we will use shorter notation for expectations, e.g. we use $\mathbb {E}[u_i(\mathbf {b})]$ instead of $\mathbb {E}_{\mathbf {b}\sim \mathbf {B}}[u_i(\mathbf {b})]$, or even $SW(\mathbf {B})$ to denote $\mathbb {E}_{\mathbf {b}\sim \mathbf {B}}[SW(\mathbf {X}(\mathbf {b}))]$.

## Combinatorial Auctions

In this section we prove an upper bound of 1.82 for the mixed price of anarchy of simultaneous all-pay auctions when bidders’ valuations are fractionally subadditive. This result improves over the previously known bound of 2 due to [23]. The proof itself might be of independent interest because we develop several structural properties of the mixed Nash equilibria. We first state our main theorem and present the key ingredients. Then we prove these ingredients in the following subsections.

### Proof Outline

Here we present a (very short) sketch of the proof highlights of the upper bound.

#### Theorem 1

The mixed PoA for simultaneous all-pay auctions with fractionally subadditive bidders is at most 1.82.

*Proof Sketch* We first illustrate the main ideas by focusing on a single item all-pay auction. W.l.o.g. we assume bidder 1 has the highest valuation $v_1$ among all bidders. First we came up with the following two lower bounds on the social welfare in equilibrium,

$$\begin{aligned} SW(\mathbf {B})\ge & {} A+\int _0^{v_1-A}1-F(x)dx,\\ SW(\mathbf {B})\ge & {} \int _0^{v_1-A}\sqrt{F(x)}dx \end{aligned}$$

where *F*(*x*) is the CDF of $\max _i\{b_i\}$ and $A=\max _x\{F_1(x)\cdot v_1-x\}$. Note that $F_1(x)$ is the CDF of $\max _{i\ne 1}\{b_i\}$. The first inequality is derived from the existing upper bound of 2 [23]. The proof of the second inequality is based on the structure of mixed Nash equilibria in all-pay auctions. By definition, we have $F_i(x)\cdot v_i-x \ge F_i(y)\cdot v_i-y$ if bidder *i* bids *x* in the Nash. By taking limits when $y\rightarrow x$, we have that $1/v_i$ equals to the derivative of $F_i$ at *x*. So $SW(\mathbf {B})$ can be rewritten as $\sum _i\int _x^{v_1}F_i(x)g_i(x)\frac{1}{F'_i(x)}dx\ge \int _x^{v_1-A}\sum _i\frac{g_i(x)}{\sum _{k\ne i}\frac{g_k(x)}{G_k(x)}}$ by using $F_i(x)=\prod _{k\ne i}G_k(x)$. Then we can adapt the following proposition to get the second lower bound for $SW(\mathbf {B})$.

#### Proposition 1

For any integer $l\ge 2$, any positive real $G_i\le 1$ and positive real $g_i$, for $1\le i\le n,$

$$\begin{aligned} \sum _{i=1}^l\frac{g_i}{\sum _{k\ne i}\frac{g_k}{G_k}} \ge \sqrt{\prod _{i=1}^lG_i} \end{aligned}$$

The bound 1.82 can be derived by an optimal convex combination of these two lower bounds for $SW(\mathbf {B})$. In order to generalize the proof from a single to multiple items, we introduce a notion, that we call expected marginal valuation denoted by $v_{ij}(x)$ for which we show that $F_{ij}(x)\cdot v_{ij}(x)-x \ge F_{ij}(y)\cdot v_{ij}(x)-y$. This allows us to treat each item separately and get the improved upper bound for simultaneous all-pay auctions.

### Full Proof

#### Proof

Given a valuation profile $\mathbf {v}=(v_1,\ldots ,v_n),$ let $\mathbf {O}= (O_1,\ldots , O_n)$ be a fixed optimal solution that maximizes the social welfare. Since $v_i$ is a fractionally subadditive valuation, let $f^{O_i}_i$ be a maximizing additive function w.r.t $O_i$. Let $j\in O_i$ be one of the items that *i* receives. We denote by $o_j$ item *j*’s contribution to the optimal social welfare, that is, $o_j=f^{O_i}_i(j)$. The optimal social welfare is thus $SW(\mathbf {O})=\sum _jo_j.$ In order to bound the price of anarchy, we consider only items with $o_j>0$, as it is without loss of generality to omit items with $o_j=0.$

For a fixed mixed Nash equilibrium $\mathbf {B},$ recall that by $F_j$ and $F_{ij}$ we denote the CDFs of the maximum bid on item *j* among all bidders, with and without the bid of bidder *i*, respectively. Observe that $F_j(x)\le F_{ij}(x)$. For any item $j\in O_i$, let $A_j=\max _{x\ge 0} \, \{F_{ij}(x)o_j-x\}.$

As a key part of the proof we use the following two inequalities that bound from below the social welfare in any mixed Nash equilibrium $\mathbf {B}$.

1 $$\begin{aligned} SW(\mathbf {B})\ge & {} \sum _{j\in [m]}\left( A_j+\int _0^{o_j-A_j}(1-F_j(x))dx\right) \end{aligned}$$

2 $$\begin{aligned} SW(\mathbf {B})\ge & {} \sum _{j\in [m]}\int _0^{o_j-A_j}\sqrt{F_j(x)}dx \end{aligned}$$

Inequality (1), suffices to provide a weaker upper bound of 2 (see [8]). The proof of Inequality (2) is much more involved, and requires deeper understanding of the properties of equilibria of the induced game. We postpone their proofs to Sect. 3.3 (Lemma 1) and Sect. 3.4 (Lemma 2) respectively.

By combining (1) and (2) we get

3 $$\begin{aligned} SW(\mathbf {B})\ge & {} \frac{1}{1+\lambda }\cdot \sum _j\left( A_j+\int _0^{o_j-A_j}(1-F_j(x))dx\right. \nonumber \\&\left. +\,\lambda \cdot \int _0^{o_j-A_j}\sqrt{F_j(x)}dx\right) , \end{aligned}$$

for any $\lambda \ge 0$. It suffices to bound from below the right-hand side of (3) with respect to the optimal social welfare. For any cumulative distribution function *F*, and any positive real number *v*, let

$$\begin{aligned} R(F,v)\mathop {=}\limits ^{\mathrm {def}}A+\int _0^{v-A}(1-F(x))dx+\lambda \cdot \int _0^{v-A}\sqrt{F(x)}dx, \end{aligned}$$

where $A=\max _{x\ge 0}\{F(x)\cdot v - x\}$. Then inequality (3) can be rewritten as $SW(\mathbf {B})\ge$ $\frac{1}{1+\lambda }\sum _jR(F_j,o_j)$. Finally, we show a lower bound on *R*(*F*, *v*) that holds for any CDF *F* and any positive real *v*.

4 $$\begin{aligned} R(F,v)\ge \frac{3+4\lambda -\lambda ^4}{6}\cdot v. \end{aligned}$$

The proof of inequality 4 is given in Sect. 3.5 (Lemma 19). Finally, we obtain that for any $\lambda > 0$,

$$\begin{aligned} SW(\mathbf {B})\ge & {} \frac{1}{1+\lambda }\sum _jR(F_j,o_j)\\\ge & {} \frac{3+4\lambda -\lambda ^4}{6\lambda +6}\cdot \sum _jo_j= \frac{3+4\lambda -\lambda ^4}{6\lambda +6}\cdot SW(\mathbf {O}) \end{aligned}$$

We conclude that the price of anarchy is at most $\frac{6\lambda +6}{3+4\lambda -\lambda ^4}\simeq 1.82$ by taking $\lambda =0.56$.

$$\square $$

### Proof of Inequality (1)

This section is devoted to the proof of the following lower bound.

#### Lemma 1

$$SW(\mathbf {B}) \ge \sum _{j\in [m]}(A_j+\int _0^{o_j-A_j}(1-F_j(x))dx).$$

#### Proof

Recall that $A_j=\max _{x\ge 0} \, \{F_{ij}(x)o_j-x\}$. We can bound bidder *i*’s utility in the Nash equilibrium $\mathbf {B}$ by $u_i(\mathbf {B})\ge \sum _{j\in O_i}A_j$. To see this, consider the deviation for bidder *i*, where he bids only for items in $O_i$, namely, for each item *j*, he bids the value $x_j$ that maximizes the expression $F_{ij}(x_j)o_j-x_j$. Since for any obtained subset $T\subseteq O_i$, he has value $v_i(T)\ge \sum _{j\in T} o_j,$ and the bids $x_j$ must be paid in any case, the expected utility with these bids is at least $\sum _{j\in O_i}\max _{x\ge 0} \,(F_{ij}(x)o_j-x)=\sum _{j\in O_i}A_j.$ With $\mathbf {B}$ being an equilibrium, we infer that $u_i(\mathbf {B})\ge \sum _{j\in O_i}A_j$.

By summing up over all bidders, we have

$$\begin{aligned} SW(\mathbf {B})= & {} \sum _{i\in [n]}u_i(\mathbf {B})+\sum _{i\in [n]}\sum _{j\in [m]}\mathbb {E}[b_{ij}] \ge \sum _{j\in [m]}A_j+\sum _{j\in [m]}\sum _{i\in [n]}\mathbb {E}[b_{ij}]\\\ge & {} \sum _{j\in [m]}\left( A_j+\mathbb {E}\left[ \max _{i\in [n]}\{b_{ij}\}\right] \right) \ge \sum _{j\in [m]}\left( A_j+\int _0^{o_j-A_j}(1-F_j(x))dx\right) . \end{aligned}$$

The first equality holds because $SW(\mathbf {B})=\sum _i\mathbb {E}_\mathbf {b}[v_i(\mathbf {b})]=\sum _{i}\mathbb {E}_\mathbf {b}[u_i(\mathbf {b})+\sum _{j\in [m]}b_{ij}].$ The second inequality follows because $\sum _{i} b_{ij}\ge \max _{i} \,b_{ij}$ and the last one is implied by the definition of the expected value of any positive random variable. $\square$

### Proof of Inequality (2)

In this section, we prove the following lemma for any mixed Nash equilibrium $\mathbf {B}$.

#### Lemma 2

$$SW(\mathbf {B}) \ge \sum _{j\in [m]}\int _0^{o_j-A_j}\sqrt{F_j(x)}dx.$$

First we show a useful lemma that holds for fractionally subadditive valuations.

#### Lemma 3

For any fractionally subadditive valuation function *v*,

$$\begin{aligned} v(S) \ge \sum _{j\in [m]}\left( v(S)-v(S{\setminus }\{j\})\right) . \end{aligned}$$

#### Proof

Let *f* be a maximizing additive function of *S* for the fractionally subadditive valuation *v*; then by definition $v(S)=f(S)$ and for every item *j* it holds that $v(S{\setminus }\{j\})\ge f(S{\setminus }\{j\})$. Then,

$$\begin{aligned} \sum _{j\in [m]}\left( v(S)-v(S{\setminus }\{j\})\right)\le & {} \sum _{j\in [m]}(f(S)-f(S{\setminus }\{j\}))\\= & {} \sum _{j\in S}f(j)=v(S). \end{aligned}$$

$$\square $$

We will use the following technical proposition.

#### Proposition 2

(restate Proposition 1) For any integer $n\ge 2$, any positive reals $G_i\le 1$ and positive reals $g_i$, for $1\le i\le n,$

$$\begin{aligned} \sum _{i=1}^n\frac{g_i}{\sum _{k\ne i}\frac{g_k}{G_k}} \ge \sqrt{\prod _{i=1}^nG_i}. \end{aligned}$$

In order to prove the proposition, we will minimize the left hand side of the inequality over all $G_i$ and $g_i$, such that

5 $$\begin{aligned} 0<G_i\le 1\quad \quad g_i>0 \quad (i\in [n]) \quad \text { where } \prod _{t=1}^n G_t \quad \text {is a constant.} \end{aligned}$$

We introduce the following notation:

$$\begin{aligned} H = \sum _{i=1}^n \frac{g_i}{\sum _{t=1,t\ne i}^n\frac{g_t}{G_t}} \qquad \text {and} \qquad \forall i \text {,} \qquad H_i = \frac{g_i}{\sum _{t=1,t\ne i}^n\frac{g_t}{G_t}}. \end{aligned}$$

Note that $H = \sum _{i=1}^n H_i.$ Our goal is to minimize *H* over all possible variables $G_i$ and $g_i$ under the constraints (5), and eventually show $H\ge \sqrt{\prod _{i=1}^nG_i}$. We also use the notation $\mathbf {G}= (G_i)_i,$ $\mathbf {g}= (g_i)_i$, $H = H(\mathbf {G},\mathbf {g})$ and $H_i = H_i(\mathbf {G},\mathbf {g})$, $\forall i$.

#### Lemma 4

For every $\mathbf {G}$ and $\mathbf {g}$ that minimize $H(\cdot ,\cdot )$ under constraints (5):

1. If  $G_i < 1$ and $G_j < 1$, then $H_i=H_j,$
2. If  $$G_i = G_j = 1$$ then $$g_i = g_j$$.

We prove Lemma 4, by proving Lemmas 5 and 6.

#### Lemma 5

Under constraints (5), if $\mathbf {G}$ and $\mathbf {g}$ minimize $H(\cdot ,\cdot )$, then for every $G_i < 1$ and $G_j <1$, $H_i(\mathbf {G},\mathbf {g}) = H_j(\mathbf {G},\mathbf {g})$.

#### Proof

For the sake of contradiction, suppose that there exist $G_i < 1$ and $G_j <1$ such that (w.l.o.g.) $H_i(\mathbf {G},\mathbf {g}) > H_j(\mathbf {G},\mathbf {g})$. Let

$$\begin{aligned} r=\min \left\{ \left( \frac{H_i(\mathbf {G},\mathbf {g})}{H_j(\mathbf {G},\mathbf {g})}\right) ^{\frac{1}{2}}, \frac{1}{G_j}\right\} . \end{aligned}$$

Notice that $$r > 1$$.

*Claim*: We claim that $H(\mathbf {G},\mathbf {g}) > H(\mathbf {G}',\mathbf {g}')$, where $\mathbf {G}' = (\frac{G_i}{r}, rG_j, \mathbf {G}_{-ij})$ and $\mathbf {g}'= (\frac{g_i}{r}, rg_j, \mathbf {g}_{-ij})$.

As usual $\mathbf {G}_{-ij}$ stands for $\mathbf {G}$ vector after eliminating $G_i$ and $G_j$ (accordingly for $\mathbf {g}_{-ij}$). Therefore $\mathbf {G}'$ and $\mathbf {g}'$ are the same as $\mathbf {G}$ and $\mathbf {g}$ by replacing $G_i$, $G_j$, $g_i$, $g_j$ by $\frac{G_i}{r}$, $rG_j$, $\frac{g_i}{r}$, $rg_j$, respectively.

*Proof of the claim*: Notice that

$$\begin{aligned} \frac{g'_i}{G'_i}=\frac{g_i/r}{G_i/r}=\frac{g_i}{G_i} \text {,} \quad \frac{g'_j}{G'_j}=\frac{rg_j}{rG_j}=\frac{g_j}{G_j} \quad \text {and} \quad \forall s \ne i,j \text {,} \quad G'_s = G_s \quad \text {and} \quad g'_s = g_s. \end{aligned}$$

Therefore, $\forall s \ne i,j$, $H_s(\mathbf {G},\mathbf {g})=H_s(\mathbf {G}', \mathbf {g}')$. So, we only need to show that $H_i(\mathbf {G},\mathbf {g})+H_j(\mathbf {G},\mathbf {g}) > H_i(\mathbf {G}',\mathbf {g}')+H_j(\mathbf {G}',\mathbf {g}')$.

$$\begin{aligned}&H_i(\mathbf {G}',\mathbf {g}')+H_j(\mathbf {G}',\mathbf {g}')\\&\quad =\frac{g'_i(x)}{\sum _{t=1,t\ne i}^n\frac{g'_t(x)}{G'_t(x)}} + \frac{g'_j(x)}{\sum _{t=1,t\ne j}^n\frac{g'_t(x)}{G'_t(x)}}\\&\quad =\frac{g_i(x)/r}{\sum _{t=1,t\ne i}^n\frac{g_t(x)}{G_t(x)}} + \frac{rg_j(x)}{\sum _{t=1,t\ne j}^n\frac{g_t(x)}{G_t(x)}}\\&\quad = \frac{H_i(\mathbf {G},\mathbf {g})}{r} + rH_j(\mathbf {G},\mathbf {g})\\&\quad = \left( \frac{1}{r}-1\right) H_i(\mathbf {G},\mathbf {g}) + (r-1)H_j(\mathbf {G},\mathbf {g}) + H_i(\mathbf {G},\mathbf {g})+H_j(\mathbf {G},\mathbf {g})\\&\quad \le \left( \frac{1}{r}-1\right) r^2H_j(\mathbf {G},\mathbf {g}) + (r-1)H_j(\mathbf {G},\mathbf {g}) + H_i(\mathbf {G},\mathbf {g})+H_j(\mathbf {G},\mathbf {g})\\&\quad = -\left( r-1\right) ^2H_j(\mathbf {G},\mathbf {g}) + H_i(\mathbf {G},\mathbf {g})+H_j(\mathbf {G},\mathbf {g})\\&\quad < H_i(\mathbf {G},\mathbf {g})+H_j(\mathbf {G},\mathbf {g}). \end{aligned}$$

In the above inequalities we used that $r>1$ and $r^2\le \frac{H_i(\mathbf {G},\mathbf {g})}{H_j(\mathbf {G},\mathbf {g})}$. The claim contradicts the assumption that $H(\mathbf {G},\mathbf {g})$ is the minimum, so the lemma holds. $\square$

#### Lemma 6

Under constraints (5), if $\mathbf {G}$ and $\mathbf {g}$ minimize $H(\cdot ,\cdot )$, then for every $G_i = G_j =1$, $g_i=g_j.$

#### Proof

For the sake of contradiction, suppose that there exist $G_i = G_j =1$ such that $g_i \ne g_j$. We will prove that for $\mathbf {g}' = (\frac{g_i+g_j}{2},\frac{g_i+g_j}{2},g_{-ij})$ (i.e. for every $k \ne i , j$, $g'_k = g_k$, and $g'_i=g'_j= \frac{g_i+g_j}{2}$), $H(\mathbf {G},\mathbf {g})> H(\mathbf {G},\mathbf {g}')$.

Notice that for every $k \ne i , j$, $H_k(\mathbf {G},\mathbf {g}') = H_k(\mathbf {G},\mathbf {g})$, since $g_i+g_j=g'_i+g'_j$ and $G_i = G_j =1$. Hence it is sufficient to show that $H_i(\mathbf {G},\mathbf {g})+H_j(\mathbf {G},\mathbf {g}) \ge H_i(\mathbf {G},\mathbf {g}')+H_j(\mathbf {G},\mathbf {g}')$. Let $A_{ij} = \sum _{t \ne j,t\ne i}\frac{g_t}{G_t}$.

$$\begin{aligned}&H_i(\mathbf {G},\mathbf {g}) + H_j(\mathbf {G},\mathbf {g}) - H_i(\mathbf {G},\mathbf {g}') - H_j(\mathbf {G},\mathbf {g}')\\&\quad = \frac{g_i}{g_j + A_{ij}} +\frac{g_j}{g_i + A_{ij}} - \frac{g_i}{\frac{g_i+g_j}{2} + A_{ij}} - \frac{g_j}{\frac{g_i+g_j}{2} + A_{ij}}\\&\quad = \frac{g_i}{g_j + A_{ij}} +\frac{g_j}{g_i + A_{ij}} - \frac{2g_i+2g_j}{g_i+g_j + 2A_{ij}} \\&\quad =g_i\frac{(g_i+A_{ij})((g_i+g_j+2A_{ij}) - 2(g_j+A_{ij}))}{(g_j + A_{ij})(g_i + A_{ij})(g_i+g_j + 2A_{ij})}\\&\qquad +\,g_j\frac{(g_j+A_{ij})((g_i+g_j+2A_{ij}) - 2(g_i+A_{ij}))}{(g_j + A_{ij})(g_i + A_{ij})(g_i+g_j + 2A_{ij})}\\&\quad =\frac{g_i(g_i+A_{ij})(g_i-g_j)+g_j(g_j+A_{ij})(g_j-g_i)}{(g_j + A_{ij})(g_i + A_{ij})(g_i+g_j + 2A_{ij})}\\&\quad =\frac{(g_i-g_j)(g^2_i - g_j^2 + A_{ij}(g_i-g_j))}{(g_j + A_{ij})(g_i + A_{ij})(g_i+g_j + 2A_{ij})}\\&\quad =\frac{(g_i-g_j)^2(g_i + g_j + A_{ij})}{(g_j + A_{ij})(g_i + A_{ij})(g_i+g_j + 2A_{ij})} > 0, \end{aligned}$$

which contradicts the assumption that $\mathbf {G}$ and $\mathbf {g}$ minimize $H(\cdot ,\cdot )$. $\square$

#### Lemma 7

If $$H_i=H_j$$, then:

1. $$g_i=g_j \Leftrightarrow G_i=G_j$$,
2. $$(g_i=rg_j>0 \text { and } r\ge 1) \Rightarrow G_i \ge r^2G_j$$.

#### Proof

Let $A_{ij} = \sum _{t \ne j,t\ne i}\frac{g_t}{G_t}$; then $H_i = \frac{g_i}{\frac{g_j}{G_j}+A_{ij}}$. By assumption:

$$\begin{aligned} \frac{g_i}{\frac{g_j}{G_j}+A_{ij}}= & {} \frac{g_j}{\frac{g_i}{G_i}+A_{ij}}\\ \frac{g_i^2}{G_i} + g_iA_{ij}= & {} \frac{g^2_j}{G_j} + g_jA_{ij}\\ (g_i- g_j)A_{ij}= & {} \frac{g^2_j}{G_j} - \frac{g_i^2}{G_i}. \end{aligned}$$

If $g_i = g_j$ then $\frac{1}{G_j} - \frac{1}{G_i}=0$, so $G_i=G_j$.

If $G_i = G_j$ then $(g_i-g_j)(g_i+g_j + A_{ij}G_i) = 0$ . Under constraints (5), $A_{ij}G_i > 0$ and $g_i,g_j> 0$, so $g_i-g_j=0$ which results in $g_i=g_j$.

If $g_i=rg_j$, with $r\ge 1$ then $(g_i- g_j)A_{ij} \ge 0$ and so $\frac{1}{G_j} - \frac{r^2}{G_i} \ge 0$, which implies $G_i \ge r^2G_j.$ $\square$

#### Lemma 8

For *n*, *k* integers, $n\ge 2$, $1\le k \le n$, $0< a\le 1$ and $g>0$:

$$\begin{aligned} L=\frac{kg}{(k-1)\frac{g}{a}+n-k}+\frac{n-k}{k\frac{g}{a}+n-k-1}\ge a. \end{aligned}$$

#### Proof

We distinguish between two cases, (1) $k > \frac{1}{1-\sqrt{a}}$ and (2) $k \le \frac{1}{1-\sqrt{a}}$.

*Case 1* ($k > \frac{1}{1-\sqrt{a}}$): For $k=n$, $L = \frac{k}{k-1}a \ge a$. We next show that $\frac{dL}{dg} \le 0$, for $n\ge 2$, $1\le k < n$, $0< a\le 1$ and $g>0$.

$$\begin{aligned} \frac{dL}{dg} = \frac{(n-k)k}{\left( \frac{(k-1)g}{a}+n-k\right) ^2}-\frac{(n-k)k}{\left( \frac{kg}{a}+n-k-1\right) ^2 a}\le & {} 0\\ \left( \frac{(k-1)g}{a}+n-k\right) ^2 - \left( \frac{kg}{a}+n-k-1\right) ^2 a\ge & {} 0\\ \left( \frac{(k-1)g}{a}+n-k - \left( \frac{kg}{a}+n-k-1\right) a^\frac{1}{2}\right)\ge & {} 0\\ \left( \frac{g}{a}\left( k-1-ka^\frac{1}{2}\right) +(n-k)\left( 1-a^\frac{1}{2}\right) +a^\frac{1}{2}\right)\ge & {} 0\\ k-1-ka^\frac{1}{2}\ge & {} 0 \end{aligned}$$

which is true by the case assumption. Therefore, *L* is non-increasing and so it is minimized for $g=\infty$. Hence, $L \ge \frac{k}{k-1}a \ge a$.

*Case 2* ($k \le \frac{1}{1-\sqrt{a}}$): *L* is minimized ($dL/dg (g^*) = 0$) for $g^* = \frac{a\left( \sqrt{a}+(n-k)\left( 1-\sqrt{a} \right) \right) }{k\sqrt{a}-k+1}$, therefore:

$$\begin{aligned} L \ge \frac{k^2\left( 1-\sqrt{a}\right) ^2+k\left( a-n\left( 1-\sqrt{a}\right) ^2-1\right) +n)}{(n-1)}, \end{aligned}$$

which is minimizes for $k = \frac{n}{2}+\frac{\left( 1+\sqrt{a}\right) }{2\left( 1-\sqrt{a}\right) }$. However, for $n\ge 2$, $\frac{n}{2}+\frac{\left( 1+\sqrt{a}\right) }{2\left( 1-\sqrt{a}\right) } \ge \frac{1}{1-\sqrt{a}}$. Notice, though, that for $k \le \frac{1}{1-\sqrt{a}}$, *L* is decreasing, so it is minimized for $k = \frac{1}{1-\sqrt{a}}$. Therefore, $L \ge \sqrt{a} \ge a$. $\square$

#### Proof

(Proposition 1)

Let $\mathbf {G}$ and $\mathbf {g}$ minimize $H(\cdot ,\cdot )$ and also let $S=\{i|G_i<1\}$ and $F=\prod _{t=1}^n G_t$. Moreover, given Lemma 4, for $g_i=\hat{g}$ for every $i \notin S$ and $j=\arg \min _{i\in S}g_i$, $H(\mathbf {G},\mathbf {g})$ can be written as:

$$\begin{aligned} H(\mathbf {G},\mathbf {g})= & {} |S|\frac{g_j}{\sum _{t \in S, t \ne j}\frac{g_t}{G_t}+(n-|S|)\hat{g}}\\&+\,(n-|S|)\frac{\hat{g}}{\sum _{t \in S}\frac{g_t}{G_t}+(n-|S|-1)\hat{g}}. \end{aligned}$$

Let $g_i = r_ig_j$, for every $i \in S$. Since $j=\arg \min _{i\in S}g_i$, then for every $i \in S$, $r_i \ge 1$. By using Lemma 7:

$$\begin{aligned} H(\mathbf {G},\mathbf {g})= & {} |S|\frac{g_j}{\sum _{t \in S, t \ne j}\frac{r_tg_j}{G_t^\frac{1}{2} G_t^\frac{1}{2}}+(n-|S|)\hat{g}}\\&+\,(n-|S|)\frac{\hat{g}}{\sum _{t \in S}\frac{r_tg_j}{G_t^\frac{1}{2} G_t^\frac{1}{2}}+(n-|S|-1)\hat{g}}\\\ge & {} |S|\frac{g_j}{\sum _{t \in S, t \ne j}\frac{g_j}{F^\frac{1}{2}}+(n-|S|)\hat{g}}\\&+\,(n-|S|)\frac{\hat{g}}{\sum _{t \in S}\frac{g_j}{F^\frac{1}{2} }+(n-|S|-1)\hat{g}}\\= & {} |S|\frac{g_j}{(|S|-1)\frac{g_j}{F^\frac{1}{2}}+(n-|S|)\hat{g}}\\&+\,(n-|S|)\frac{\hat{g}}{|S|\frac{g_j}{F^\frac{1}{2} }+(n-|S|-1)\hat{g}}. \end{aligned}$$

Let $$g = \frac{g_j}{\hat{g}}$$, then:

$$\begin{aligned} H(\mathbf {G},\mathbf {g}) \ge \frac{|S|g}{(|S|-1)\frac{g}{F^\frac{1}{2}}+n-|S|}+\frac{n-|S|}{|S|\frac{g}{F^\frac{1}{2} }+n-|S|-1}. \end{aligned}$$

If $|S|=0$, $H(\mathbf {G},\mathbf {g}) \ge \frac{n}{n-1} \ge 1 \ge \sqrt{F}$. else, due to Lemma 8, $H(\mathbf {G},\mathbf {g}) \ge \sqrt{F}$. $\square$

We are now ready to proceed with the proof of Lemma 2. Recall that $o_j$ is the contribution of item *j* to the optimum social welfare. If player *i* is the one receiving item *j* in the optimum allocation, then $A_j = \max _{x\ge 0} \{F_{ij}(x)\cdot o_j-x\}$. The proof of Lemma 2 needs a careful technical preparation that we divided into a couple of lemmas.

First of all, we define the expected marginal valuation of item *j* for player *i*. For given mixed strategy $B_i,$ the distribution of bids on items in $[m]\setminus \{j\}$ depends on the bid $b_{ij},$ so one can consider the given conditional expectation:

#### Definition 4

Given a mixed bidding profile $\mathbf {B}=(B_1, B_2, \ldots , B_n)$, the expected marginal valuation $v_{ij}(x)$ of item *j* for player *i* when $b_{ij}=x$ is defined as

$$\begin{aligned} v_{ij}(x)\mathop {=}\limits ^{\mathrm {def}}\mathop {\mathbb {E}}\limits _{\mathbf {b}\sim \mathbf {B}}[v_i(X_i(\mathbf {b})\cup \{j\})-v_i(X_i(\mathbf {b}){\setminus }\{j\})|b_{ij}=x]. \end{aligned}$$

For a given $\mathbf {B}$, let $\varphi _{ij}(x)$ denote the probability that bidder *i* gets item *j* when she bids *x* on item *j*. It is clear that $\varphi _{ij}$ is non-decreasing and $\varphi _{ij}(x)\le F_{ij}(x)$ (they are equal when no ties occur).

#### Lemma 9

For a given $\mathbf {B}$, for any bidder *i*, item *j* and bids $x\ge 0$ and $y\ge 0$,

$$\begin{aligned} \varphi _{ij}(y)\cdot v_{ij}(x)=\mathop {\mathbb {E}}\limits _{\mathbf {b}\sim \mathbf {B}}\left[ v_i(X_i(\mathbf {b}'))-v_i(X_i(\mathbf {b}'){\setminus }\{j\})|b_{ij}=x\right] , \end{aligned}$$

where $\mathbf {b}'$ is the modified bid of $\mathbf {b}$ such that $\mathbf {b}'=\mathbf {b}$ except that $b'_{ij}=y$.

#### Proof

$$\begin{aligned}&\mathop {\mathbb {E}}\limits _{\mathbf {b}\sim \mathbf {B}}[v_i(X_i(\mathbf {b}'))-v_i(X_i(\mathbf {b}'){\setminus }\{j\})|b_{ij}=x]\\= & {} \mathop {\mathbb {E}}\limits _{\mathbf {b}\sim \mathbf {B}}[v_i(X_i(\mathbf {b}'))-v_i(X_i(\mathbf {b}'){\setminus }\{j\})|b_{ij}=x, j \in X_i(\mathbf {b}')]\mathrm {Pr}(j \in X_i(\mathbf {b}')|b_{ij}=x)\\&+\, \mathop {\mathbb {E}}\limits _{\mathbf {b}\sim \mathbf {B}} [v_i(X_i(\mathbf {b}'))-v_i(X_i(\mathbf {b}'){\setminus }\{j\})|b_{ij}=x, j \notin X_i(\mathbf {b}')]\mathrm {Pr}(j \notin X_i(\mathbf {b}')|b_{ij}=x)\\= & {} \mathop {\mathbb {E}}\limits _{\mathbf {b}\sim \mathbf {B}}[v_i(X_i(\mathbf {b}'))-v_i(X_i(\mathbf {b}'){\setminus }\{j\})|b_{ij}=x, j \in X_i(\mathbf {b}')]\mathrm {Pr}(j \in X_i(\mathbf {b}')|b_{ij}=x)\\= & {} \mathop {\mathbb {E}}\limits _{\mathbf {b}\sim \mathbf {B}} [v_i(X_i(\mathbf {b}'))-v_i(X_i(\mathbf {b}'){\setminus }\{j\})|b_{ij}=x, j \in X_i(\mathbf {b}')]\cdot \varphi _{ij}(y)\\= & {} \mathop {\mathbb {E}}\limits _{\mathbf {b}\sim \mathbf {B}} [v_i(X_i(\mathbf {b}')\cup \{j\})-v_i(X_i(\mathbf {b}'){\setminus }\{j\})|b_{ij}=x, j \in X_i(\mathbf {b}')]\cdot \varphi _{ij}(y)\\= & {} \mathop {\mathbb {E}}\limits _{\mathbf {b}\sim \mathbf {B}} [v_i(X_i(\mathbf {b}')\cup \{j\})-v_i(X_i(\mathbf {b}'){\setminus }\{j\})|b_{ij}=x]\cdot \varphi _{ij}(y)\\= & {} \varphi _{ij}(y)\cdot v_{ij}(x). \end{aligned}$$

The second equality is due to $\mathbb {E}_{\mathbf {b}\sim \mathbf {B}}[v_i(X_i(\mathbf {b}')))-v_i(X_i(\mathbf {b}'){\setminus }\{j\})|b_{ij}=x, j\notin X_i(\mathbf {b}')]=0;$ the third one holds because $b'_{ij}=y,$ and that other players’ bids have distribution $\times _{k\ne i} B_k.$ The fourth one is obvious, since $X_i(\mathbf {b}')=X_i(\mathbf {b}')\cup \{j\}$ given that $j\in X_i(\mathbf {b}').$ The last two equalities follow from the fact that $v_i(X_i(\mathbf {b}')\cup \{j\})-v_i(X_i(\mathbf {b}'){\setminus }\{j\})$ is independent of the condition $j\in X_i(\mathbf {b}')$ and of the player *i*’s bid on item *j*. $\square$

#### Definition 5

Given a Nash equilibrium $\mathbf {B}$, we say a bid *x* is *good for bidder i and item j* (or $b_{ij}=x$ is *good*) if $\mathbb {E}[u_i(\mathbf {b})]=\mathbb {E}[u_i(\mathbf {b})|b_{ij}=x]$, otherwise we say $b_{ij}=x$ is *bad*.

#### Lemma 10

Given a Nash equilibrium $\mathbf {B}$, for any bidder *i* and item *j*, $\mathrm {Pr}[b_{ij}\text { is bad}]=0$.

#### Proof

The lemma follows from the definition of Nash equilibrium; otherwise we can replace the bad bids with good bids and improve the bidder’s utility. $\square$

#### Lemma 11

Given a Nash equilibrium $\mathbf {B}$, for any bidder *i*, item *j*, good bid *x* and any bid $y\ge 0$,

$$\begin{aligned} \varphi _{ij}(x)\cdot v_{ij}(x)-x \ge \varphi _{ij}(y)\cdot v_{ij}(x)-y. \end{aligned}$$

Moreover, for a good bid $x>0,$ $\varphi _{ij}(x)>0$ holds.

#### Proof

Let $\mathbf {b}'$ be the modified bid of $\mathbf {b}$ such that $\mathbf {b}'=\mathbf {b}$ except that $b'_{ij}=y$.

$$\begin{aligned} \mathbb {E}[u_i(\mathbf {b})]= \mathbb {E}[u_i(\mathbf {b})|b_{ij}=x]\ge \mathbb {E}[u_i(\mathbf {b}')|b_{ij}=x]. \end{aligned}$$

Now we consider the difference between the above two terms:

$$\begin{aligned} 0\le & {} \mathbb {E}[u_i(\mathbf {b})|b_{ij}=x]-\mathbb {E}[u_i(\mathbf {b}')|b_{ij}=x]\\= & {} \mathbb {E}[v_i(X_i(\mathbf {b}))-b_{ij}|b_{ij}=x]-\mathbb {E}[v_i(X_i(\mathbf {b}'))-b'_{ij}|b_{ij}=x]\\= & {} \mathbb {E}[v_i(X_i(\mathbf {b}))-v_i(X_i(\mathbf {b}){\setminus }\{j\})|b_{ij}=x]-\mathbb {E}[v_i(X_i(\mathbf {b}'))\\&-\,v_i(X_i(\mathbf {b}'){\setminus }\{j\}|b_{ij}=x]+y-x\\= & {} (\varphi _{ij}(x)\cdot v_{ij}(x)-x)-(\varphi _{ij}(y)\cdot v_{ij}(x)-y). \end{aligned}$$

The second equality holds since $X_i(\mathbf {b}){\setminus }\{j\}=X_i(\mathbf {b}'){\setminus }\{j\};$ the third equality holds by Lemma 9.

Finally, $\varphi _{ij}(x)>0$ for positive good bids follows by taking $y=0,$ since with $\varphi _{ij}(x)=0$ the left hand side of the inequality would be negative. $\square$

Next, by using the above lemma, we are able to show several structural results for Nash equilibria.

#### Definition 6

Given a mixed strategy profile $\mathbf {B}$, we say that a positive bid $x>0$ is in bidder *i*’s *support on item* *j*, if for all $\varepsilon >0$, $G_{ij}(x)-G_{ij}(x-\varepsilon )>0$.

#### Lemma 12

Given a mixed strategy profile $\mathbf {B}$, if a positive bid *x* is in bidder *i*’s support on item *j*, then for every $\varepsilon >0$, there exists $x-\varepsilon < x' \le x$ such that $x'$ is good.

#### Proof

Suppose on the contrary that there is an $\varepsilon >0$ such that for all $x'$, such that $x-\varepsilon < x' \le x$, $x'$ is bad. Then $\mathrm {Pr}[b_{ij}\text { is bad}]\ge G_{ij}(x)-G_{ij}(x-\varepsilon )>0$ (given that *x* is in the support), which contradicts Lemma 10. $\square$

#### Lemma 13

Given a Nash equilibrium $\mathbf {B}$, if $x>0$ is in bidder *i*’s support on item *j*, then there must exist another bidder $k\ne i$ such that *x* is also in the bidder *k*’s support on item *j*, i.e. for all $\varepsilon >0$, $G_{kj}(x)-G_{kj}(x-\varepsilon )>0$.

#### Proof

Assume on the contrary that for each player $k \ne i$, there exists $\varepsilon _k>0$ such that $G_{kj}(x)-G_{kj}(x-\varepsilon _k)=0$. Clearly, for $\varepsilon =\min \{\varepsilon _k|k\ne i\}$ it holds that $G_{kj}(x)-G_{kj}(x-\varepsilon )=0$ for all bidders $k\ne i.$ That is $\varphi _{ij}(x)=\varphi _{ij}(x-\varepsilon )$. By Lemma 12, there exists $x-\varepsilon < x' \le x$ such that $x'$ is good for player *i*. Since $\varphi _{ij}$ is a non-decreasing function and $\varphi _{ij}(x)=\varphi _{ij}(x-\varepsilon )$, we have $\varphi _{ij}(x')=\varphi _{ij}(x-\varepsilon )$. By Lemma 11, $\varphi _{ij}(x')\cdot v_{ij}(x')-x' \ge \varphi _{ij}(x-\varepsilon )\cdot v_{ij}(x')-x+\varepsilon$ which contradicts the fact that $\varphi _{ij}(x')=\varphi _{ij}(x-\varepsilon )$ and $x'>x-\varepsilon$. $\square$

#### Lemma 14

Given a Nash equilibrium $\mathbf {B}$, for bidder *i* and item *j*, there are no $x>0$ such that $\mathrm {Pr}[b_{ij}=x]>0$, i.e. there are no mass points in the bidding strategy, except for possibly 0.

#### Proof

Assume on the contrary that there exists a bid $x>0$ such that $\mathrm {Pr}[b_{ij}=x]>0$ for some bidder *i* and item *j*. By Lemma 10, *x* is good for bidder *i* and item *j*, and $\varphi _{ij}(x)>0$ by Lemma 11.

According to Lemma 13, there must exist a bidder *k* such that *x* is in her support on item *j*. We can pick a sufficiently small $\varepsilon$ such that $\varepsilon <(x-\varepsilon )\cdot \varphi _{ij}(x)\cdot \mathrm {Pr}[b_{ij}=x].$ This can be done since $(x-\varepsilon )$ increases when $\varepsilon$ decreases. Due to Lemma 12 there exists $x-\varepsilon < x' \le x$ such that $x'$ is good for bidder *k* and item *j*. Now we consider the following two cases for $x'.$

*Case 1* $v_{kj}(x')\le x'$. Then $\varphi _{kj}(x')\cdot v_{kj}(x')-x'\le \varphi _{kj}(x')\cdot x'-x'\le (1-\varphi _{ij}(x)\cdot \mathrm {Pr}[b_{ij}=x])\cdot x' - x'<0,$ contradicting Lemma 11. The first inequality holds by the case assumption. The second holds because player *k* cannot get item *j* with bid $x'$ whenever player *i* gets it by bidding *x*. The last inequality holds because both $\varphi _{ij}(x)>0$ and $\mathrm {Pr}[b_{ij}=x]>0.$

*Case 2* $v_{kj}(x')> x'$. Then there exists a sufficiently small $\varepsilon '$ such that $\varepsilon '\le (x-\varepsilon )\cdot \varphi _{ij}(x)\cdot \mathrm {Pr}[b_{ij}=x]-\varepsilon$. So $\varepsilon +\varepsilon '\le x'\cdot \varphi _{ij}(x)\cdot \mathrm {Pr}[b_{ij}=x]$. Then,

$$\begin{aligned}&\varphi _{kj}(x+\varepsilon ')\cdot v_{kj}(x')-x-\varepsilon '\\&\ge (\varphi _{kj}(x')+\varphi _{ij}(x)\cdot \mathrm {Pr}[b_{ij}=x])\cdot v_{kj}(x')-x-\varepsilon '\\&> \varphi _{kj}(x')\cdot v_{kj}(x')+\varphi _{ij}(x)\cdot \mathrm {Pr}[b_{ij}=x]\cdot x'-x'-(x-x')-\varepsilon '\\&> \varphi _{kj}(x')\cdot v_{kj}(x')+\varphi _{ij}(x)\cdot \mathrm {Pr}[b_{ij}=x]\cdot x'-x'-\varepsilon -\varepsilon '\\&\ge \varphi _{kj}(x')\cdot v_{kj}(x')-x', \end{aligned}$$

which contradicts Lemma 11. Here the first inequality holds because the probability that player *k* gets the item with bid $x+\varepsilon '$ is at least the probablity that he gets it by bidding $x'$ plus the probability that *i* bids *x* and gets the item (these two events for $\mathbf {b}_{-k}$ are disjoint). The second inequality holds by case assumption, and the rest hold by our assumptions on $\varepsilon$ and $\varepsilon '.$ $\square$

#### Lemma 15

Given a Nash equilibrium $\mathbf {B}$, for any bidder *i* and item *j*, $\varphi _{ij}(x)=F_{ij}(x)$ for all $x>0$.

#### Proof

The lemma follows immediately from Lemma 14. The probablity that some player $k\ne i$ bids exactly *x* is zero. Thus $F_{ij}(x)$ equals the probability that the highest bid of players other than *i* is strictly smaller than *x*,  and $1-F_{ij}(x)$ is the probability that it is strictly higher. Therefore $\varphi _{ij}(x)=F_{ij}(x).$ $\square$

#### Lemma 16

Given a Nash equilibrium $\mathbf {B}$, for any bidder *i*, item *j* and good bids $x_1>x_2>0$, $v_{ij}(x_1)\ge v_{ij}(x_2)$.

#### Proof

By Lemma 11, we have $(\varphi _{ij}(x_1)-\varphi _{ij}(x_2))\cdot v_{ij}(x_1)\ge x_1-x_2$ and $(\varphi _{ij}(x_2)-\varphi _{ij}(x_1))\cdot v_{ij}(x_2)\ge x_2-x_1$. Combining these two inequalities, we have

$$\begin{aligned} \frac{1}{v_{ij}(x_1)}\le \frac{\varphi _{ij}(x_1)-\varphi _{ij}(x_2)}{x_1-x_2}\le \frac{1}{v_{ij}(x_2)}. \end{aligned}$$

$$\square $$

#### Lemma 17

Given a Nash equilibrium $\mathbf {B}$ and item *j*, let $T=\sup \{x|x$ is in some bidder’s support on item $j\}$. For any bid $x<T$, *x* is in some bidder’s support on item *j*.

#### Proof

Assume on the contrary that there exist a bid $x<T$ such that *x* is not in any bidder’s support. Then there exists $\delta >0$ such that $G_{ij}(x)=G_{ij}(x-\delta )$ for all bidder *i*. Let $y=\sup \{z|\forall i, G_{ij}(x)=G_{ij}(z)\}$. By Lemma 14, $G_{ij}$ is continuous. So we have $G_{ij}(y)=G_{ij}(x)=G_{ij}(x-\delta )$ for any bidder *i*. That is $F_{ij}(y)=F_{ij}(x-\delta )$ for any bidder *i*.

By the definition of supremum, there exists a bidder *k* such that for any $\varepsilon >0$, $G_{kj}(y+\varepsilon )>G_{kj}(x)=G_{kj}(y)$. By Lemma 10, there exists a good bid $y^+\in (y,y+\varepsilon ]$ for bidder *k* and item *j*. We pick a sufficiently small $\varepsilon$ such that $(F_{kj}(y^+)-F_{kj}(y))\cdot v_{kj}(y^+)<\delta$. This can be done since $F_{kj}$ is continuous by Lemma 14 and $v_{kj}$ is non-decreasing by Lemma 16.

$$\begin{aligned}&F_{kj}(x-\delta )\cdot v_{ij}(y^+)-x+\delta \\&=F_{ij}(y)\cdot v_{ij}(y^+)-x+\delta \\&>F_{ij}(y)\cdot v_{ij}(y^+)-y^++\delta \\&>F_{ij}(y^+)\cdot v_{ij}(y^+)-y^+, \end{aligned}$$

which contradicts Lemmas 11 and 15. $\square$

#### Lemma 18

Given a Nash equilibrium $\mathbf {B}$, if $x>0$ is a good bid for bidder *i* and item *j*, and $F_{ij}$ is differentiable in *x*,  then

$$\begin{aligned} \frac{1}{v_{ij}(x)}=\frac{dF_{ij}(x)}{dx}. \end{aligned}$$

#### Proof

Notice that $v_{ij}(x)\ne 0$ by Lemma 11. By Lemmas 11 and 15, we have $F_{ij}(x)\cdot v_{ij}(x)-x \ge F_{ij}(y)\cdot v_{ij}(x)-y$ for all $y\ge 0$. So for any $\varepsilon >0$,

$$\begin{aligned} F_{ij}(x)\cdot v_{ij}(x)-x\ge & {} F_{ij}(x+\varepsilon )\cdot v_{ij}(x)-x-\varepsilon \\ F_{ij}(x)\cdot v_{ij}(x)-x\ge & {} F_{ij}(x-\varepsilon )\cdot v_{ij}(x)-x+\varepsilon . \end{aligned}$$

That is,

$$\begin{aligned} \frac{F_{ij}(x+\varepsilon )-F_{ij}(x)}{\varepsilon }\le & {} \frac{1}{v_{ij}(x)}, \\ \frac{F_{ij}(x)-F_{ij}(x-\varepsilon )}{\varepsilon }\ge & {} \frac{1}{v_{ij}(x)}. \end{aligned}$$

The lemma follows by taking the limit when $\varepsilon$ goes to 0. $\square$

#### Proof of Lemma 2

Since $G_{ij}(x)$ is non-decreasing, continuous (Lemma 14) and bounded by 1, $G_{ij}(x)$ is differentiable on almost all points. That is, the set of all non-differentiable points has Lebesgue measure 0. So it will not change the value of integration if we remove these points. Therefore it is without loss of generality to assume $G_{ij}(x)$ is differentiable for all *x*. Let $g_{ij}(x)$ be the derivative of $G_{ij}(x)$, i.e. probability density function for bidder *i*’s bidding on item *j*. Using Lemma 3, we have

$$\begin{aligned} SW(\mathbf {B})&=\sum _i\mathbb {E}[v_i(X_i(\mathbf {b}))]\\&\ge \sum _i\sum _j\mathbb {E}[v_i(X_i(\mathbf {b}))-v_i(X_i(\mathbf {b}){\setminus }\{j\})]\\&\ge \sum _i\sum _j\int _{0}^{o_j-A_j}\mathbb {E}[v_i(X_i(\mathbf {b}))-v_i(X_i(\mathbf {b}){\setminus }\{j\})|b_{ij}=x]\cdot g_{ij}(x)dx\\&\ge \sum _i\sum _j\int _0^{o_j-A_j}F_{ij}(x)\cdot v_{ij}(x)\cdot g_{ij}(x)dx. \end{aligned}$$

The second inequality follows by the law of total probability, and the third is due to Lemmas 9 and 15. By Lemma 18 and the fact that $F_{ij}(x)=\prod _{k\ne i}G_{kj}(x)$, if *x* is good, $g_{ij}(x)>0$ and $G_{ij}(x)>0$ we have for all *j*

$$\begin{aligned} F_{ij}(x)\cdot v_{ij}(x)\cdot g_{ij}(x)=&\frac{F_{ij}(x)\cdot g_{ij}(x)}{\frac{dF_{ij}}{dx}(x)}\\ =\frac{\prod _{k\ne i}G_{kj}(x)\cdot g_{ij}(x)}{\sum _{k\ne i}\left( g_{kj}\cdot \prod _{s\ne k \wedge s\ne i}G_{sj}\right) } =&\frac{g_{ij}(x)}{\sum _{k\ne i}\frac{g_{kj}(x)}{G_{kj}(x)}}. \end{aligned}$$

By concentrating on a specific item *j*, let $S_x$ be the set of bidders so that *x* is in their support. We next show that $|S_x| \ge 2$ for all $x\in (0, o_j-A_j]$. Recall that $A_j=\max _x \,\{F_{ij}(x)\cdot o_j-x\}$ for the bidder *i* who receives *j* in $\mathbf {O}.$ Let $h_{ij}=\min \{ x | F_{ij}=1\}$ (we use minimum instead of infimum, since, by Lemma 14, $F_{ij}$ is continuous). By definition $h_{ij}$ should be in some bidder’s support. Moreover, $A_j \ge F_{ij}(h_{ij})\cdot o_j-h_{ij} = o_j-h_{ij}$, resulting in $o_j - A_j \le h_{ij}$. Therefore, by Lemma 17, for all $x\in (0, o_j-A_j]$, $\,x$ is in some bidder’s support and by Lemma 13, there are at least 2 bidders such that *x* is in their supports.

By the definition of derivative, for all $i\not \in S_x$, $g_{ij}(x)=0$. Similarly, we have $g_{ij}(x)>0$ and $G_{ij}(x)>0$ for all $i\in S_x$ by definition 6. Moreover, for every $i \in S_x$, *x* is good for bidder *i* and item *j*, since *x* is in their support. So, for any fixed $x\in (0, o_j-A_j],$ $\sum _{i\in [n]} F_{ij}(x)\cdot v_{ij}(x)\cdot g_{ij}(x)= \sum _{i\in S_x} F_{ij}(x)\cdot v_{ij}(x)\cdot g_{ij}(x),$ and according to Proposition 1,

$$\begin{aligned} \sum _{i\in [n]} F_{ij}(x)\cdot v_{ij}(x)\cdot g_{ij}(x)\ge & {} \sum _{i\in S_x}\frac{g_{ij}(x)}{\sum _{k\ne i, k\in S_x}\frac{g_{kj}}{G_{kj}}}\\\ge & {} \sqrt{\prod _{i\in S_x}G_{ij}(x)}\ge \sqrt{\prod _{i\in [n]}G_{ij}(x)}. \end{aligned}$$

Merging all these inequalities,

$$\begin{aligned} SW(\mathbf {B})\ge \sum _{j\in [m]}\int _0^{o_j-A_j}\sqrt{\prod _{i\in [n]}G_{ij}(x)}dx=\sum _{j\in [m]}\int _0^{o_j-A_j}\sqrt{F_j(x)}dx. \end{aligned}$$

$$\square $$

### Proof of Inequality (4)

In this section we prove the following technical lemma.

#### Lemma 19

For any CDF *F* and any real $v>0$, $R(F,v)\ge \frac{3+4\lambda -\lambda ^4}{6}v$.

In order to obtain a lower bound for *R*(*F*, *v*) as stated in the lemma, we show first that we can restrict attention to cumulative distribution functions of a simple special form, since these constitute worst cases for *R*(*F*, *v*). In the next lemma, for an arbitrary CDF *F* we will define a simple piecewise linear function ${\hat{F}}$ that satisfies the following two properties.

$$\begin{aligned} \int _0^{v-A}(1-{\hat{F}}(x))dx= & {} \int _0^{v-A}(1-F(x))dx \text { and } \int _0^{v-A}\sqrt{{\hat{F}}(x)}dx\\\le & {} \int _0^{v-A}\sqrt{F(x)}dx. \end{aligned}$$

Once we establish this, it will be convenient to lower bound $R({\hat{F}},v)$ for the given type of piecewise linear functions ${\hat{F}}.$

#### Lemma 20

For any CDF *F* and real $v>0$, there always exists another CDF ${\hat{F}}$ such that $R(F,v)\ge R({\hat{F}},v)$ that is defined by

$$\begin{aligned} {\hat{F}}(x)=\left\{ \begin{array}{cl} 0 &{} \text {if } x \in [0, x_0] \\ \frac{x+A}{v} &{} \text {if } x \in (x_0, v-A] \end{array} \right. \end{aligned}$$

where $$A=\max _{x\ge 0}\{F(x)\cdot v - x\}$$.

#### Proof

First notice that $\max _{x\ge 0}\{\hat{F}(x)\cdot v - x\} = A$. By the definition of Riemann integration, we can represent the integration as the limit of Riemann sums. For any positive integer *l*, let $R_l$ be the Riemann sum if we partition the interval $[0,v-A]$ into small intervals of size $(v-A)/l$. That is

$$\begin{aligned} R_l(F,v)=A+\frac{v-A}{l}\cdot \left( \sum _{i=0}^{l-1}(1-F(x_i))+\lambda \cdot \sum _{i=0}^{l-1}\sqrt{F(x_i)}\right) \end{aligned}$$

where $x_i=\frac{i}{l} \cdot (v-A)$. So we have $R(F,v)=\lim _{l\rightarrow \infty }R_l(F,v)$.

For any given *l*, let $i^*$ be the index such that $\sum _{i>i^*}(x_i+A)/v < \sum _{i=0}^{l-1}F(x_i)$ and $\sum _{i>=i^*}(x_i+A)/v \ge \sum _{i=0}^{l-1}F(x_i)$. We define ${\hat{F}}_l$ as follows.

$$\begin{aligned} {\hat{F}}_l(x)=\left\{ \begin{array}{cl} 0 &{} \text {if } x<x_{i^*}\\ \sum \nolimits _{i=0}^{l-1}F(x_i)-\sum \nolimits _{i>i^*}(x_i+A)/v &{} \text {if } x\in [x_{i^*},x_{i^*+1})\\ (x+A)/v &{} \text {if } x\in [x_{i^*+1} ,v-A] \end{array} \right. \end{aligned}$$

**Fig. 1:**
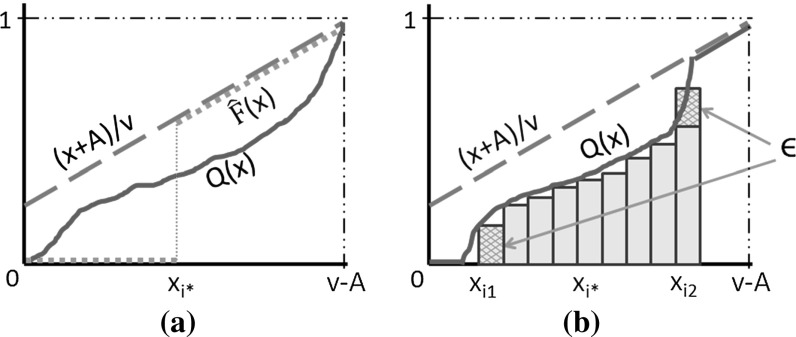
Figure **a** illustrates ${\hat{F}}(x)=\lim _{l\rightarrow \infty }{\hat{F}}_l(x)$ and figure **b** shows how $Q'$ is derived from *Q*

It is straight-forward to check that ${\hat{F}}(x) =\lim _{l\rightarrow \infty }{\hat{F}}_l(x)$, as described in the statement of the lemma. We will show that for any *l*, $R_l(F,v)\ge R_l({\hat{F}}_l,v)$. Then the lemma follows by taking the limit, since $R_l(F,v)\rightarrow R(F,v),$ and $R_l({\hat{F}},v)\rightarrow R({\hat{F}},v).$ Figure 1a illustrates ${\hat{F}}(x)$ (when we take the limit of *l* to infinity).

By the construction of ${\hat{F}}_l$, it is easy to check that $\sum _{i=0}^{l-1}F(x_i)=\sum _{i=0}^{l-1}{\hat{F}}_l(x_i)$ and $\max _x\{{\hat{F}}_l(x)\cdot v-x\}= A$. Then in order to prove $R_l(F,v)\ge R_l({\hat{F}}_l,v)$, it is sufficient to prove that $\sum _{i=0}^{l-1}\sqrt{F(x_i)}\ge \sum _{i=0}^{l-1}\sqrt{{\hat{F}}_l(x_i)}$. Let $\mathcal {Q}$ be the set of CDF functions such that $\forall Q\in \mathcal {Q}$, $\sum _{i=0}^{l-1}Q(x_i)=\sum _{i=0}^{l-1}F(x_i)$ and $A = \max _{x\ge 0} \{Q(x)\cdot v - x\}$, meaning further that $Q(x)\le (x+A)/v$, for all $x\ge 0$. We will show that ${\hat{F}}_l(x)$ has the minimum value for the expression $\sum _{i=0}^{l-1}\sqrt{\hat{F}_l(x_i)}$ within $\mathcal {Q}$.

Assume on the contrary that some other function $Q\in \mathcal {Q}$ has the minimum value for $\sum _{i=0}^{l-1}\sqrt{Q(x_i)}$ within $\mathcal {Q}$ and $Q(x_{j})\ne {\hat{F}}_l(x_j)$ for some $x_j$. Let $i_1$ be the smallest index such that $Q(x_{i_1})>0$ and $i_2$ be the largest index such that $Q(x_{i_2})<(x_{i_2}+A)/v$. By the monotonicity of *Q*, we have $i_1\le i_2$. Due to the assumption that $Q(x_j)\ne {\hat{F}}_l(x_j)$ for some $x_j$ and $\sum _{i=0}^{l-1}\sqrt{Q(x_i)}\le \sum _{i=0}^{l-1}\sqrt{{\hat{F}}_l(x_i)}$, we get $i_1\ne i_2$. So $i_1<i_2$ and $Q(x_{i_1})<Q(x_{i_2})$ by the monotonicity of CDF functions. Now consider another CDF function $Q'$ such that $Q'(x_i)=Q(x_i)$ for all $i\ne i_1 \wedge i\ne i_2$, $Q'(x_{i_1})=Q(x_{i_1})-\epsilon$ and $Q'(x_{i_2})=Q(x_{i_2})+\epsilon$ where $\epsilon = \min \{Q(x_{i_1}), (x_{i_2}+A)/v-Q(x_{i_2})\}$. Figure 1b shows how we modify *Q* to $Q'$. It is easy to check $Q'\in \mathcal {Q}$ and $\sum _{i=0}^{l-1}\sqrt{Q(x_i)}>\sum _{i=0}^{l-1}\sqrt{Q'(x_i)}$ which contradicts the optimality of *Q*. The inequality holds because of $\sqrt{a}+\sqrt{b}>\sqrt{a-c}+\sqrt{b+c}$ for all $0<c<a<b$, which can be proved by simple calculations. $\square$

Now we are ready to proceed with the proof of Lemma 19.

#### Proof of Lemma 19

By Lemma 20, for any fixed $v>0$, we only need to consider the CDF’s that have the following form. For any positive *A* and $x_0$ such that $x_0+A\le v$,

$$\begin{aligned} F(x)=\left\{ \begin{array}{ll} 0 &{} \quad \text {if } x \in [0, x_0] \\ \frac{x+A}{v} &{}\quad \quad \text {if } x \in (x_0, v-A] \end{array}\right. \end{aligned}$$

Clearly, $\max _{x\ge 0}\{F(x)\cdot v-x\}=A$. Let $t=\frac{A+x_0}{v}$. Then

$$\begin{aligned} R(F,v)= & {} A+\int _0^{v-A}1-F(x)dx+\lambda \cdot \int _0^{v-A}\sqrt{F(x)}dx\\= & {} v-\frac{v}{2}\cdot \left( \frac{x+A}{v}\right) ^2\bigg \vert ^{v-A}_{x0} +\lambda \cdot \frac{2v}{3}\cdot \left( \frac{x+A}{v}\right) ^{\frac{3}{2}}\bigg |^{v-A}_{x_0}\\= & {} v-\frac{v}{2}\cdot (1-t^2)+\lambda \cdot \frac{2v}{3}\cdot (1-t^{\frac{3}{2}})\\= & {} v\cdot \left( \frac{1}{2}(1+t^2)+\frac{2\lambda }{3}(1-t^{\frac{3}{2}})\right) \end{aligned}$$

By optimizing over *t*, the above formula is minimized when $t=\lambda ^2\le 1$. That is,

$$\begin{aligned} R(F,v)\ge v\cdot \left( \frac{1}{2}(1+\lambda ^4)+\frac{2\lambda }{3}(1-\lambda ^3)\right) =\frac{3+4\lambda -\lambda ^4}{6}\cdot v \end{aligned}$$

$$\square $$

## Multi-Unit Auctions

In this section, we propose a randomized all-pay mechanism for the multi-unit setting, where *m* identical items are to be allocated to *n* bidders. Markakis and Telelis [18] and de Keijzer et al. [14] have studied the price of anarchy for several multi-unit auction formats. The current best upper bound obtained was 1.58 for mixed Nash equilibria.

We propose a *randomized* all-pay mechanism that induces a *unique pure* Nash equilibrium, with an improved price of pnarchy bound of 4 / 3. We call the mechanism Random proportional-share allocation mechanism (PAM), as it is a randomized version of Kelly’s celebrated proportional-share allocation mechanism for divisible resources [15]. The mechanism works as follows (illustrated as Mechanism 1).
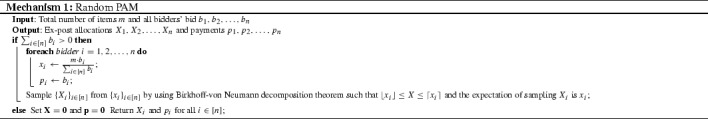


Each bidder submits a non-negative real $b_i$ to the auctioneer. After soliciting all the bids from the bidders, the auctioneer associates a real number $x_i$ with bidder *i* that is equal to $x_i=\frac{m\cdot b_i}{\sum _{i\in [n]}b_i}$. Each player pays their bid, $p_i=b_i$. In the degenerate case, where $\sum _ib_i=0$, then $x_i=0$ and $p_i=0$ for all *i*.

We turn the $x_i$’s to a random allocation as follows. Each bidder *i* secures $\lfloor x_i \rfloor$ items and gets one more item with probability $x_i-\lfloor x_i \rfloor$. An application of the Birkhoff-von Neumann decomposition theorem [4] guarantees that given an allocation vector $(x_1,x_2,\dots ,x_n)$ with $\sum _ix_i=m$, one can always find a randomized allocation^1^ with random variables $X_1,X_2,\dots ,X_n$ such that $\mathbb {E}[X_i]\!=\!x_i$ and $\mathrm {Pr}[\lfloor x_i \rfloor \!\le \! X_i \le \lceil x_i \rceil ]\!=\!1$.

We next show that the game induced by the Random PAM when the bidders have submodular valuations is *isomorphic* to the game induced by Kelly’s mechanism for a single divisible resource when bidders have piece-wise linear concave valuations. For convenience, we review the definition of isomorphism between games as appears in Monderer and Shapley [19].

### Definition 7

[19]. Let $\varGamma _1$ and $\varGamma _2$ be games in strategic form with the same set of players [*n*]. For $k=1,2$, let $(A^i_k)_{i\in [n]}$ be the strategy sets in $\varGamma _k$, and let $(u^i_k)_{i\in [n]}$ be the utility functions in $\varGamma _k$. We say that $\varGamma _1$ and $\varGamma _2$ are isomorphic if there exists bijections $\phi ^i:a^i_1\rightarrow a^i_2$, $i\in [n]$ such that for every $i\in [n]$ and every $(a^1,a^2,\dots , a^n)\in \times _{i\in [n]}A^i_1$,

$$\begin{aligned} u^i_1(a^1,a^2,\dots ,a^n)=u^i_2(\phi ^1(a^1),\phi ^2(a^2),\dots ,\phi ^n(a^n)). \end{aligned}$$

### Theorem 2

Any game induced by the Random PAM applied to the multi-unit setting with submodular bidders is *isomorphic* to a game induced from Kelly’s mechanism applied to a single divisible resource with piece-wise linear concave functions.

### Proof

For each bidder *i*’s submodular valuation function $f_i:\{0,1,\ldots ,m\} \rightarrow R^+$, we associate a concave function $g_i:[0,1] \rightarrow R^+$ such that,

6 $$\begin{aligned} \text {for every } x \in [0,m], \quad g_i(x/m)=f_i(\lfloor x \rfloor )+(x-\lfloor x \rfloor )\cdot (f_i(\lfloor x \rfloor +1)-f_i(\lfloor x \rfloor )).\nonumber \\ \end{aligned}$$

**Fig. 2:**
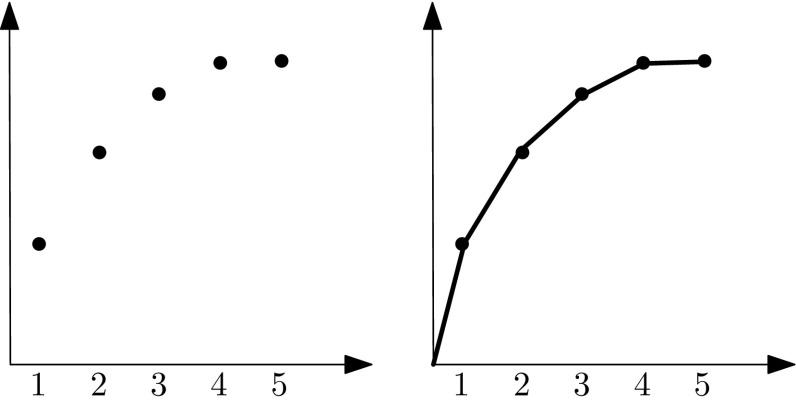
Illustration of the concave function. The *left part* of the figure depicts some submodular function *f*, while the *right part* depicts the modified concave function *g*. One can verify that *g* is concave if *f* is submodular

Essentially, $g_i$ is the piecewise linear function that comprises the line segments that connect $f_i(k)$ with $f_i(k+1)$, for all nonnegative integers *k*. It is easy to see that $g_i$ is concave if $f_i$ is submodular (see also Fig. 2 for an illustration).

We use identity functions as the bijections $\phi ^i$ of Definition 7. Therefore, it suffices to show that, for any pure strategy profile $\mathbf {b}$, $u_i(\mathbf {b})=u'_i(\mathbf {b})$, where $u_i$ and $u'_i$ are the bidder *i*’s utility functions in the first and second game, respectively. Let $x_i=\frac{m\cdot b_i}{\sum _ib_i}$, then

$$\begin{aligned} u_i(\mathbf {b})= & {} (x_i-\lfloor x_i \rfloor )f_i(\lfloor x_i \rfloor +1) + (1-x_i+\lfloor x_i \rfloor )f_i(\lfloor x_i \rfloor )-b_i\\= & {} f_i(\lfloor x_i \rfloor ) + (x_i-\lfloor x_i \rfloor )(f_i(\lfloor x_i \rfloor +1)-f_i(\lfloor x_i \rfloor ))-b_i\\= & {} g_i\left( \frac{x_i}{m}\right) - b_i = g_i\left( \frac{b_i}{\sum _ib_i}\right) - b_i = u'_i(\mathbf {b}), \end{aligned}$$

where $g_i\left( \frac{b_i}{\sum _ib_i}\right) - b_i$ is the utility of player *i*, under strategy profile $\mathbf {b}$, in Kelly’s mechanism. $\square$

Given submodular functions $(f_i)_i$, let $(g_i)_i$ be the associated concave functions as defined in (6). We can show the following equivalence between optimal welfares.

### Lemma 21

The optimum social welfare in the multi-unit setting, with submodular valuations $\mathbf {f}= (f_1,\ldots , f_n)$, is equal to the optimal social welfare in the divisible resource allocation with concave valuations $\mathbf {g}= (g_1,\ldots g_n)$, where $\mathbf {g}$ is derived from $\mathbf {f}$ as described in (6).

### Proof

For any valuation profile $\mathbf {v}$ and (randomized) allocation $\mathcal {A}$, we denote by $SW_{\mathbf {v}}(\mathcal {A})$ the social welfare of allocation $\mathcal {A}$ under the valuations $\mathbf {v}$. For any fractional allocation $\mathbf {x}=(x_1,\ldots , x_n)$, such that $\sum _ix_i = m$, let $\mathbf {X}(\mathbf {x}) = (X_1(\mathbf {x}),\ldots , X_n(\mathbf {x}))$ be the random allocation as computed by the Random PAM given the fractional allocation $\mathbf {x}$. Also let $\mathbf {o}=(o_1, \ldots , o_n)$ and $\mathbf {O}= (O_1, \ldots , O_n)$ be the optimal allocations in the divisible resource allocation problem and in the multi-unit auction, respectively.

First we show that $SW_{\mathbf {g}}(\mathbf {o}) \ge SW_{\mathbf {f}}(\mathbf {O})$. Consider the fractional allocation $\mathbf {o}' = (o'_1, \ldots , o'_n)$, where $o'_i = O_i/m$, for every *i*. Then it is easy to see that for every *i*, $g_i(o'_i) = f_i(\lfloor O_i \rfloor )+(O_i-\lfloor O_i \rfloor )\cdot (f_i(\lfloor O_i \rfloor +1)-f_i(\lfloor O_i \rfloor )) = f_i({O_i})$, since $O_i$ is an integer. Therefore, $SW_{\mathbf {g}}(\mathbf {o}) \ge SW_{\mathbf {g}}(\mathbf {o}') = SW_{\mathbf {f}}(\mathbf {O})$, by the optimality of $\mathbf {o}$.

Now we show $SW_{\mathbf {f}}(\mathbf {O}) \ge SW_{\mathbf {g}}(\mathbf {o})$. Note that for any fractional allocation $\mathbf {x}$, such that $\sum _jx_j = m$, $\mathbb {E}_{\mathbf {X}(\mathbf {x})} [f_i({X_i(\mathbf {x})})] = f_i(\lfloor x_i \rfloor )+(x_i-\lfloor x_i \rfloor )\cdot (f_i(\lfloor x_i \rfloor +1)-f_i(\lfloor x_i \rfloor )) = g_i(x_i/m)$, for every *i*. By the optimality of $\mathbf {O}$, $SW_{\mathbf {f}}(\mathbf {O}) \ge \mathbb {E}_{\mathbf {X}(m\cdot \mathbf {o})}[SW_{\mathbf {f}}(\mathbf {X}(m\cdot \mathbf {o}))] = SW_{\mathbf {g}}(\mathbf {o})$. $\square$

Theorem 2 and Lemma 21, allow us to obtain the existence and uniqueness of the pure Nash equilibrium, as well as the price of anarchy bounds of Random PAM by the corresponing results on Kelly’s mechanism for a single divisible resource [13]. Moreover, it can be shown that there are no other mixed Nash equilibria by adopting the arguments of [5] for Kelly’s mechanism. The main conclusion of this section is summarized in the following Corollary.

### Corollary 1

Random PAM induces a *unique pure* Nash equilibrium when applied to the multi-unit setting with submodular bidders. Moreover, the price of anarchy of the mechanism is *exactly* 4 / 3.

## Single Item Auctions

In this section, we study mixed Nash equilibria in a single item all-pay auction. First, in Sect. 5.1 we measure the inefficiency of mixed Nash equilibria, showing tight results for the price of anarchy. Then in Sect. 5.2, we analyze the quality of two other important criteria, the *expected revenue (the sum of bids)* and the quality of the expected *highest submission (the maximum bid)*, which is a standard objective in crowdsourcing contests [6]. For these objectives, we show a lower bound of $v_2/2$, where $v_2$ is the second highest value among all bidders’ valuations. In the following, we drop the word expected while referring to the revenue or to the maximum bid.

We quantify the loss of revenue and the highest submission in the worst-case equilibria. We show that the all-pay auction achieves a 2-approximation comparing to the conventional procurement (modeled as the first price auction), when considering worst-case mixed Nash equilibria; we show in Sect. 5.3 that the revenue and the maximum bid of the conventional procurement equals $v_2$ in the worst case. We also consider other structures of rewards allocation and conclude that allocating the entire reward to the highest bidder is the only way to guarantee the approximation factor of 2. Roughly speaking, allocating all the reward to the top prize is the optimal way to maximize the maximum bid and revenue among all the prior-free all-pay mechanisms where the designer has no prior information about the participants’ skills.

Throughout this section we assume that the players are ordered based on decreasing order of their valuations, i.e. $v_1 \ge v_2 \ge \cdots \ge v_n$.

### Social Welfare

Our analysis is based on the characterization of the Nash equilibrium with single item by [1]. En route, we also show the price of anarchy is 8 / 7 for auctions with two players.

#### Theorem 3

The mixed price of anarchy of single item all-pay auction is at most 1.185.

#### Proof

Based on the results of [1], inefficient Nash equilibria only exist when players’ valuations are in the form $v_1>v_2=\cdots =v_k>v_{k+1}\ge \cdots \ge v_n$ (with $v_2>0$), where players $k+1$ through *n* bid zero with probability 1. W.l.o.g., we assume that $v_1=1$ and $v_i=v>0$, for $2\le i \le k$. Let $P_1$ be the probability that bidder 1 gets the item in any such mixed Nash equilibrium denoted by $\mathbf {B}$. Then the expected utility of bidder 1 in $\mathbf {b}\sim \mathbf {B}$ can be expressed by $\mathbb {E}[u_1(\mathbf {b})]=P_1\cdot 1 -\mathbb {E}[b_1]$. Based on the characterization in [1], no player would bid above *v* in any Nash equilibrium and nobody bids exactly *v* with positive probability. Therefore, if player 1 deviates to *v*, she will gets the item with probability 1. By the definition of Nash equilibrium, we have $\mathbb {E}[u_1(\mathbf {b})] \ge \mathbb {E}[u_1(v,\mathbf {b}_{-i})]=1-v$, resulting in $P_1 \ge 1-v + \mathbb {E}[b_1]$.

It has been shown in the proof of Theorem 2C in [1], that $\mathbb {E}[b_1]$ is minimized when players 2 through *k* play symmetric strategies. Following their results, we can extract the following equations (for a specific player *i*):

$$\begin{aligned} G_1(x)= & {} \frac{x}{v \prod _{i'\ne 1,i}{G_{i'}(x)}}, \quad \forall x \in (0,v], \\ \prod _{i'\ne 1}{G_{i'}(x)}= & {} 1-v+x, \quad \forall x \in (0,v] \end{aligned}$$

recall that $G_{i'}(x)$ is the CDF according to which player $i'$ bids in $\mathbf {B}$. Since players 2 through *k* play symmetric strategies, $G_{i'}(x)$ should be identical for $i'\ne 1$. Then, for some $i' \ne 1$,

$$\begin{aligned} G_1(x)=\frac{x}{v\cdot G_{i'}^{k-2}(x)}=\frac{x}{v\cdot \left( 1-v+x\right) ^{\frac{k-2}{k-1}}}, \qquad \forall x \in (0,v] \end{aligned}$$

Note that $1-v+x \le 1$, and so we get $G_1(x) \le \frac{x}{v \left( 1-v+x\right) }$ (for two players, $G_1(x) = \frac{x}{v }$) and

$$\begin{aligned} \mathbb {E}[b_1] \ge \int _0^v \left( 1-\frac{x}{v \left( 1-v+x\right) }\right) dx = v-1-\frac{(1-v)\ln (1-v)}{v}. \end{aligned}$$

Now we can derive that $P_1 \ge \frac{1-v}{v}\ln \frac{1}{1-v}.$ For two players, $\mathbb {E}[b_1]=\int _0^v \left( 1-x/v \right) dx=v/2$ and so $P_1 = 1-v/2$.

The expected social welfare in $\mathbf {B}$ is $\mathbb {E}[SW(b)] \ge P_1+(1-P_1)v \ge \frac{(1-v)^2}{v}\ln \frac{1}{1-v} + v$. The expression, $T(v) = \frac{(1-v)^2}{v}\ln \frac{1}{1-v} + v$, is minimized for $v\approx 0.5694$ and therefore, the price of anarchy is at most $T(0.5694) \approx 1.185$. Particularly, for two players, $\mathbb {E}[SW(b)] \ge 1-v/2+v^2/2$, which is minimized for $v=1/2$ and therefore the price of anarchy for two players is at most 8 / 7. $\square$

#### Theorem 4

The mixed price of anarchy of single item all-pay auction is at least 1.185.

#### Proof

Consider *n* players, with valuations $v_1=1$ and $v_i=v>0$, for $2\le i\le n$. Let $\mathbf {B}$ be the Nash equilibrium, where bidders bid according to the following CDFs,

$$\begin{aligned} G_1(x)= & {} \frac{x}{v \left( 1-v+x\right) ^\frac{n-2}{n-1}} \;\;\; x\in [0,v], \\ G_i(x)= & {} \left( 1-v+x\right) ^\frac{1}{n-1} \;\;\; x\in [0,v],\;\;\; i\ne 1 \end{aligned}$$

Note that $F_i(x) = \prod _{i'\ne i} G_{i'}(x)$ is the probability of bidder *i* getting the item when she bids *x*, for every bidder *i*.

$$\begin{aligned} F_1(x)= & {} (1-v+x)\qquad x\in [0,v], \\ F_i(x)= & {} \frac{x}{v} \qquad x\in [0,v], \;\;\;i \ne 1. \end{aligned}$$

If player 1 bids any value $x \in [0,v]$, her utility is $u_1 = F_1(x) \cdot 1 -x = 1-v$. Bidding greater than *v* is dominated by bidding *v*. If any player $i\ne 1$ bids any value $x\in [0,v]$, her utility is $u_i = F_i(x) \cdot v -x = 0$. Bidding greater than *v* results in negative utility. Hence, $\mathbf {B}$ is a Nash equilibrium. Let $P_1$ be the probability that bidder 1 gets the item in $\mathbf {B}$, then

$$\begin{aligned} \mathbb {E}[SW(b)]= & {} 1\cdot P_1+(1-P_1)v = v+(1-v)P_1 \\= & {} v+(1-v)\int _0^v G_i^{n-1}(x) dG_1(x). \end{aligned}$$

When *n* goes to infinity, $\mathbb {E}[SW(b)]$ converges to $v+(1-v)\int _0^v \frac{1-v}{v(1-v+x)}dx = v+(1-v)\frac{1-v}{v}\ln {\frac{1}{1-v}}=\frac{(1-v)^2}{v}\ln \frac{1}{1-v} + v = T(v)$. If we set $v=0.5694$, the price of anarchy is at least $T(v) \approx 1.185$.

For $n=2$, $\mathbb {E}[SW(b)] = v+(1-v) \int _0^v\frac{1-v+x}{v} = v+(1-v)(1-v/2) = 1-v/2+v^2/2$, which for $v=1/2$ results in price of anarchy at least 8 / 7. $\square$

### Revenue and Maximum Bid

In this section we bound the revenue and the maximum bid of the single-item all-pay auction, for the case of mixed Nash equilibria. Specifically, the revenue and the maximum bid have value of at least $v_2/2$ and this value goes to $v_2/2$ when the number of bidders goes to infinity and $v_2/v_1$ approaches 0.

#### Theorem 5

In any mixed Nash equilibrium of the single-item all-pay auction, the revenue and the maximum bid are at least half of the second highest valuation.

#### Proof

Let *k* be any integer greater or equal to 2, such that $v_1 \ge v_2 = \cdots = v_k \ge v_{k+1} \ge \cdots \ge v_n$. Let $F(x) = \prod _i G_i(x)$ be the CDF of the maximum bid *h*. By the characterization of [1], in any mixed Nash equilibrium, players with valuation less than $v_2$ do not participate (always bid zero) and there exist two players 1, *i* bidding continuously in the interval $[0,v_2]$. Then, by [1], $F_1 = (v_1-v_2+x)/{v_1}$ and $F_{i}(x)=x/ v_2$, for any $x \in (0,v_2]$. Therefore, we get

$$\begin{aligned} F(x) = F_{i}(x) G_{i}(x)= \frac{x}{v_2} G_{i}(x). \end{aligned}$$

In the proof of Theorem 2C in [1], it is argued that $G_{i_1}(x)$ is maximized (and therefore the expected maximum bid is minimized) when all the *k* players play symmetrically (except for the first player, in the case that $v_1 > v_2$). So, *F*(*x*) is maximized for $G_{i}= \left( \prod _{i'\ne 1} G_{i'}\right) ^{\frac{1}{k-1}} = F_1^{\frac{1}{k-1}} = \left( \frac{v_1-v_2+x}{v_1} \right) ^{\frac{1}{k-1}}$. Finally we get

$$\begin{aligned} E[h]= & {} \int _0^\infty (1-F(x))dx \ge \int _0^{v_2}\left( 1-\frac{x}{v_2}\left( \frac{v_1-v_2+x}{v_1} \right) ^{\frac{1}{k-1}}\right) dx \\\ge & {} v_2 - \int _0^{v_2}\frac{x}{v_2}dx = \frac{1}{2} v_2. \end{aligned}$$

The same lower bound also holds for the expected revenue, which is at least as high as the expected maximum bid. This lower bound is tight for the expected maximum bid, as indicated by our analysis, when *k* goes to infinity and for the symmetric mixed Nash equilibrium. In the next lemma, we show that this lower bound is also tight for the expected revenue. $\square$

#### Lemma 22

There exists a mixed Nash equilibrium of the single-item all-pay auction, where the revenue converges to $v_2/2$ when the number of players goes to infinity and $v_2/v_1$ approaches 0.

#### Proof

In [1], the authors provide results for the revenue in all possible equilibria. For the case that $v_1 = v_2$, the expected revenue is always equal to $v_2$. To show a tight lower bound, we consider the case where $v_1 > v_2$ and there exist *k* players with valuation $v_2$ playing symmetrically in the equilibrium, letting *k* go to infinity. For this case, based on [1], the revenue is equal to^2^

$$\begin{aligned} \sum _i\mathbb {E}[b_i] = v^2+\left( 1-{v}\right) \mathbb {E}[b_1], \end{aligned}$$

where, $\mathbb {E}[b_1] = \int _0^{v} \left( 1-G_1(x)\right) dx$. From the proof of Theorem 5 we can derive that $G_1(x) = F(x)/F_1(x) = \frac{x}{v}\left( {1-v+x} \right) ^{\frac{1}{k-1}-1} = \frac{x}{v}\left( {1-v+x} \right) ^{-1}$, when *k* goes to infinity. By substituting we get,

$$\begin{aligned} \sum _i\mathbb {E}[b_i]= & {} v^2+\left( 1-{v}\right) \int _0^{v}\left( 1-\frac{x}{v}\left( {1-v+x} \right) ^{-1}\right) dx\\= & {} v^2+\left( 1-{v}\right) \left( v-\frac{1}{v} \left( v + (1-v)\ln (1-v)\right) \right) \\= & {} 2v - 1 - \frac{(1-v)^2}{v}\ln (1-v)\\= & {} v - (1-v)\left( 1 + \frac{1-v}{v}\ln (1-v)\right) . \end{aligned}$$

By taking limits, we finally derive that $\lim _{v \rightarrow 0} \left( \frac{\sum _i\mathbb {E}[b_i]}{v}\right) = 1/2$. $\square$

Finally, the next theorem indicates that allocating the entire reward to the highest bidder is the best choice. In particular a prior-free all-pay mechanism is presented by a probability vector $\mathbf {q}= (q_i)_{i\in [n]}$, with $\sum _{i\in [n]} q_i = 1$, where $q_i$ is the probability that the $i^{th}$ highest bidder is allocated the item, for every $i \le n$.

#### Theorem 6

For any prior-free all-pay mechanism that assigns the item to the highest bidder with probability strictly less than 1, i.e. $q_1<1$, there exists a valuation profile and mixed Nash equilibrium such that the revenue and the maximum bid are strictly less than $v_2/2$.

#### Proof

We will assert the statement of the theorem for the valuation profile $(1, v, 0,0,\dots ,0)$, where $v\in (0,1)$ is the second highest value. It is safe to assume that $q_2\in [0,q_1)$ 3. We show that the following bidding profile is a mixed Nash equilibrium. The first two bidders bid on the interval $[0,v(q_1-q_2)]$ and the other bidders bid 0. The CDF of bidder 1’s bid is $G_1(x)=\frac{x}{v(q_1-q_2)}$ and the CDF of bidder 2’s bid is $G_2(x)=x/(q_1-q_2)+1-v$. It can be checked that this is a mixed Nash equilibrium by the following calculations. For every bid $x\in [0,v(q_1-q_2)]$,

$$\begin{aligned} u_1(x)= & {} G_2(x)\cdot q_1+(1-G_2(x))\cdot q_2-x= q_1-v(q_1-q_2) \\ u_2(x)= & {} G_1(x)\cdot q_1v+(1-G_1(x))\cdot q_2v-x= q_2v \end{aligned}$$

The expected revenue is

$$\begin{aligned}&\int _{0}^{v(q_1-q_2)}(1-G_1(x))dx + \int _{0}^{v(q_1-q_2)}(1-G_2(x))dx\\&\quad =\int _{0}^{v(q_1-q_2)}\left( 1-\frac{x}{v(q_1-q_2)}\right) dx + \int _{0}^{v(q_1-q_2)}\left( 1-\left( \frac{x}{q_1-q_2}+1-v\right) \right) dx\\&\quad =\frac{v(q_1-q_2)}{2}+\frac{v^2(q_1-q_2)}{2} \end{aligned}$$

When *v* goes to 0, the revenue go to $v(q_1-q_2)/2<v/2$ since $q_1-q_2<1$. Obviously, the same happens with the maximum bid, which is at most the same as the revenue. $\square$

### Conventional Procurement

In this section we give bounds on the expected revenue and maximum bid of the single-item first-price auction. In the following, we just write revenue and maximum bid instead of expected revenue and expected maximum bid, respectively.

#### Theorem 7

In any mixed Nash equilibrium, the revenue and the maximum bid lie between the two highest valuations. There further exists a tie-breaking rule, such that in the worst-case, these quantities match the second highest valuation (This can also be achieved, under the no-overbidding assumption).

#### Lemma 23

In any mixed Nash equilibrium, if the expected utility of any player *i* with valuation $v_i$ is 0, then with probability 1 the maximum bid is at least $v_i$.

#### Proof

Consider any mixed Nash equilibrium $\mathbf {b}\sim \mathbf {B}$ and let $h=\max _i \{b_i\}$ be the highest bid; *h* is a random variable induced by $\mathbf {B}$. For the sake of contradiction, assume that *h* is *strictly* less than $v_i$ with probability $p>0$. Then, there exists $\varepsilon >0$ such that $h < v_i-\varepsilon$ with probability *p*. Consider now the deviation of player *i* to pure strategy $s_i = v_i - \varepsilon$. $s_i$ would be the maximum bid with probability *p* and therefore the utility of player *i* would be at least $p(v_i-(v_i-\varepsilon ))=p\cdot \varepsilon >0$. This contradicts the fact that $\mathbf {B}$ is an equilibrium and completes the proof of lemma. $\square$

#### Lemma 24

In any mixed Nash equilibrium, if *v* is the highest valuation, any player with valuation strictly less than *v* has expected utility equal to 0.

#### Proof

In [8] (Theorem 5.4), they proved that the price of anarchy of mixed Nash equilibria, for the single-item first-price auction, is exactly 1. This means that the player(s) with the highest valuation gets the item with probability 1. Therefore, any player with valuation strictly less than *v* gets the item with zero probability and hence, her expected utility is 0. $\square$

Consider the players ordered based on their valuations so that $v_1 \ge v_2 \ge v_3 \ge \cdots \ge v_n$. In order to prove Theorem 7, we distinguish between two cases: i) $v_1 >v_2$ and ii) $v_1 = v_2$.

#### Lemma 25

If $v_1>v_2$, the maximum bid of any mixed Nash equilibrium, is at least $v_2$ and at most $v_1$. If we further assume no-overbidding, the maximum bid is exactly $v_2$.

#### Proof

If $v_1>v_2$, by Lemma 24, the expected utility of player 2 equals 0. From Lemma 23, the highest bid is at least $v_2$ with probability 1. Moreover, if there exist players bidding above $v_1$ with positive probability, then at least one of them (whoever gets the item with positive probability) would have negative utility for that bid and would prefer to deviate to 0; so, the bidding profile couldn’t be an equilibrium. Therefore, the maximum bid lies between $v_1$ and $v_2$.

If we further assume no-overbidding, nobody, apart from player 1, would bid above $v_2$. So, the same hold for player 1, who has an incentive to bid arbitrarily close to $v_2$.

$$\square $$

#### Corollary 2

If $v_1>v_2$, there exists a tie breaking rule, under which the maximum bid of the worst-case mixed Nash equilibrium is exactly $v_2$.

#### Proof

Due to Lemma 25, it is sufficient to show a tie breaking rule, where there exists a mixed Nash equilibrium with highest bid equal to $v_2$. Consider the tie-breaking rule where, in a case of a tie with player 1 (the bidder of the highest valuation), the item is always allocated to player 1. Under this tie-breaking rule, the pure strategy profile, where everybody bids $v_2$ is obviously a pure Nash equilibrium, with $v_2$ being the maximum bid. $\square$

#### Lemma 26

If $v_1=v_2$, the maximum bid of any mixed Nash equilibrium, equals $v_2$.

#### Proof

Consider a set *S* of $k \ge 2$ players having the same valuation $v_1=v_2=\cdots = v_k = v$ and the rest having a valuation strictly less than *v*. For any mixed Nash equilibrium $\mathbf {b}\sim \mathbf {B}$ and any player *i*, let $G_i$ and $F_i$ be the CDFs of $b_i$ and $\max _{i' \ne i} b_{i'}$, respectively. We define $l_i = \inf \{x|G_i(x) > 0\}$ to be the infimum value of player’s *i* support in $\mathbf {B}$. We would like to prove that $\max _i l_i = v$. For the sake of contradiction, assume that $\max _i l_i < v$ (Assumption 1).

We next prove that, under Assumption 1, $l_i=l$ for any player $i \in S$ and for some $0 \le l < v$. We will assume that $l_{j} < l_i$ for some players $i,j \in S$ (Assumption 2) and we will show that Assumption 2 contradicts Assumption 1. There exists $\varepsilon >0$ such that $l_{j}+\varepsilon < l_i$. Moreover, based on the definition of $l_{j}$, for any $\varepsilon ' >0$, $G_j(l_j+\varepsilon ') > 0$ and so $G_j(l_j+\varepsilon ) > 0$. When player’s *j* bid is derived by the interval $[l_j,l_j+\varepsilon ]$, she receives the item with zero probability, since $l_i > l_{j}+\varepsilon$. Therefore, for any bid of her support that is at most $l_{j}+\varepsilon$, her utility is zero ($G_j(l_j+\varepsilon ) > 0$, so there should be such a bid). Since $\mathbf {B}$ is a mixed Nash equilibrium, her total expected utility should also be zero. In that case, Lemma 23 contradicts Assumption 1, and therefore Assumption 2 cannot be true (under Assumption 1). Thus, for any player $i \in S$, $l_i=l$ for some $0 \le l < v$.

Moreover, Lemma 24 indicates that no player $i \notin S$ bids above *l* with positive probability, i.e. $G_i(l)=1$ for all $i \notin S$. We now show that for any $i \in S$, $G_i$ cannot have a mass point at *l*, i.e. $G_i(l)=0$ for all $i \in S$.

*Case 1.* If $G_i(l) > 0$ for all *i*, then $p = \prod _i G_i(l) > 0$ is the probability that the highest bid is *l*, or more precisely, it is the probability that all players in *S* bid *l* and a tie occurs. Given that this event occurs, there exists a player $j \in S$ that gets the item with probability $p_j$ strictly less than 1 (this is the conditional probability). Therefore, player *j* has an incentive to deviate from *l* to $l+\varepsilon$, for $\varepsilon < (1-p_j)(v-l)$ (so that $p_j(v-l)< v-(l+\varepsilon )$); this contradicts the fact that $\mathbf {B}$ is an equilibrium.

*Case 2.* If $G_i(l) > 0$ and $G_j(l)=0$ for some $i,j \in S$, then *l* is in the support of player *i*, but she does never receives the item when she bids *l*, since player *j* bids above *l* with probability 1. Therefore, the expected utility of player *i* is 0 and due to Lemma 23 this cannot happen under Assumption 1.

Overall, we have proved so far that, under Assumption 1 (that now has become $l < v$), $G_i(l)=0$ for all $i \in S$ and $G_i(l)=1$ for all $i \notin S$. Since $k \ge 2$, $F_i(l)=\prod _{i'\ne i} G_{i'}(l) = 0$ for all *i*. Consider any player $i\in S$ and let $u_i$ be her expected utility. Based on the definition of $l_i$, for any $\varepsilon >0$, there exists $x(\varepsilon ) \in [l,l+\varepsilon ]$, such that $x(\varepsilon )$ is in the support of player *i*. Therefore, $u_i \le F_i(x(\varepsilon ))(v-x(\varepsilon )) \le F_i(l+\varepsilon )(v-l)$. As $F_i$ is a CDF, it should be right-continuous and so for any $\delta > 0$, there exists some $\varepsilon > 0$, such that $F_i(l+\varepsilon )(v-l) < \delta$ and therefore, $u_i < \delta$. We can contradict Assumption 1, right away by using Lemma 23, but we give a bit more explanation. Assume that, in $\mathbf {B}$, the maximum bid *h* is strictly less than *v* with probability $p>0$. Then, there exists some $\varepsilon ' >0$, such that $h < v-\varepsilon '$ with probability *p*. If we consider any $\delta < p(v-\varepsilon ')$, it is straight forward to see that player *i* has an incentive to deviate to the pure strategy $v-\varepsilon '$. Therefore, we showed that Assumption 1 cannot hold and so the highest bid is at least *v* with probability 1. Similar to the proof of Lemma 25, nobody will bid above *v* in any mixed Nash equilibrium. $\square$

## Footnotes

1 As an example, assume $x_1=2.5, x_2=1.6, x_3=1.9$. One can define a random allocation such that assignments (3, 2, 1), (3, 1, 2) and (2, 2, 2) occur with probabilities 0.1, 0.4, and 0.5 respectively.

2 For simplicity we assume $v_1=1$ and $v_2=v$.

3 Otherwise, consider the tie-breaking rule that allocates the item equiprobably. Then for $q_2 \ge q_1$, the strategy profile where all players bid zero is strictly dominant.
